# Rydberg-Stark deceleration of atoms and molecules

**DOI:** 10.1140/epjti/s40485-015-0028-4

**Published:** 2016-03-01

**Authors:** Stephen D. Hogan

**Affiliations:** Department of Physics and Astronomy, University College London, Gower Street, London, WC1E 6BT UK

**Keywords:** Rydberg states of atoms and molecules, Stark effect, Stark deceleration, Cold atoms and molecules

## Abstract

The large electric dipole moments associated with highly excited Rydberg states of atoms and molecules make gas-phase samples in these states very well suited to deceleration and trapping using inhomogeneous electric fields. The methods of Rydberg-Stark deceleration with which this can be achieved are reviewed here. Using these techniques, the longitudinal motion of beams of atoms and molecules moving at speeds as high as 2500 m/s have been manipulated, with changes in kinetic energy of up to |*Δ* *E*_kin_|=1.3×10^−20^ J (|*Δ* *E*_kin_|/*e*=80 meV or |*Δ* *E*_kin_|/*h* *c*=650 cm ^−1^) achieved, while decelerated and trapped samples with number densities of 10^6^– 10^7^ cm ^−3^ and translational temperatures of ∼150 mK have been prepared. Applications of these samples in areas of research at the interface between physics and physical chemistry are discussed.

## Introduction

### Rydberg states of atoms and molecules

**Fig. 1:**
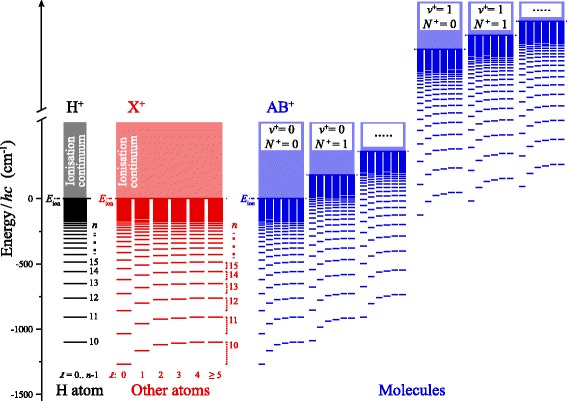
Rydberg states of atoms and molecules. Schematic diagram of series of Rydberg states in the hydrogen atom (*H*), in other non-hydrogenic atoms (*X*), and in molecules (*AB*)

All atoms and molecules possess Rydberg states. These are excited electronic states of high principal quantum number, *n*, that form series converging to each quantum state (electronic, vibrational, rotational, spin-orbit or hyperfine) of the atomic or molecular ion core (see Fig. 1). To first order the energies of these states are given by the Rydberg formula [1]

1 $$\begin{array}{@{}rcl@{}} E_{n\ell} = E_{\text{ion}} - \frac{hc\,R_{M}}{(n-\delta_{\ell})^{2}}, \end{array} $$

where *E*_ion_ is the energy associated with the Rydberg series limit, *R*_*M*_=*R*_*∞*_ *μ*_red_/*m*_e_ is the Rydberg constant corrected for the reduced mass, *μ*_red_=*M* *m*_e_/(*M*+*m*_e_), of the atom or molecule for which *M* is the mass of the ion core and *m*_e_ is the electron mass, *δ*_*ℓ*_ is a quantum defect which is dependent on the orbital angular momentum quantum number, *ℓ*, of the Rydberg electron, and *h* and *c* are the Planck constant and speed of light in vacuum, respectively.

Rydberg states converging to the lowest ionisation limit of atoms decay via spontaneous emission. The corresponding fluorescence lifetimes of the shortest-lived low- *ℓ* states typically exceed 1 *μ*s for values of *n*>30. In molecules low- *ℓ* Rydberg states often predissociate on timescales ≪1 *μ*s before spontaneous emission can occur [2, 3]. However, for molecules with a stable ion core, provided the Rydberg electron possesses sufficient orbital angular momentum (typically if *ℓ*≥4) predissociation cannot occur directly, and long-lived states with characteristic properties similar to those of Rydberg states in the H atom result.

In non-hydrogenic atoms and in molecules, low- *ℓ* Rydberg states generally posses non-zero quantum defects, i.e. *δ*_*ℓ*_>0. These arise because the Rydberg electron penetrates the non-spherically symmetric, incompletely screened, ion core to which it is bound. As a result these states, for which *ℓ*≲4, are more tightly bound than higher- *ℓ* states with the same values of *n*, for which *δ*_*ℓ*_≃0 [see Eq. (1) and Fig. 1]. Consequently, for each value of *n* Rydberg states with *ℓ*≥4 can, to a good approximation, be considered degenerate in energy in the same way that all states in the H atom, or other hydrogenic atoms (e.g., D, T, He ^+^, Li ^2+^, $\overline {\mathrm {H}}$ and Ps), are.

The *ℓ*-degeneracy of hydrogenic Rydberg states leads to linear Stark energy shifts, *E*_Stark_, in an electric field, $\vec {F}$ [4]. These linear Stark shifts can be expressed in the form

2 $$\begin{array}{@{}rcl@{}} E_{\text{Stark}} = -\vec{\mu}_{\text{elec}}\cdot\vec{F}, \end{array} $$

and are therefore a consequence of each state possessing an electric dipole moment, $\vec {\mu }_{\text {elec}}$. For each value of *n*, the maximum induced electric dipole moment *μ*_max_≃(3/2) *n* ^2^ *e* *a*_0_, where *e* is the electron charge and *a*_0_ is the Bohr radius corrected for the reduced mass and the charge of the ion core [5]. These electric dipole moments exceed 1000 D for *n*>16, and make states with high values of *n* particularly sensitive to electric fields [6]. Furthermore, in the presence of inhomogeneous fields forces, $\vec {F}$, can be exerted on atoms or molecules in such states where

3 $$\begin{array}{@{}rcl@{}} \vec{f} &=& -\nabla\,E_{\text{Stark}}\\ &=& \phantom{-}\nabla\left(\vec{\mu}_{\text{elec}}\cdot\vec{F}\right). \end{array} $$

From the dependence of these forces on the relative orientation of the electric dipole moment and electric field vectors, states with dipole moments oriented parallel (antiparallel) to the field exhibit negative (positive) Stark energy shifts. Therefore in inhomogeneous fields atoms or molecules in these states are forced toward higher-field (lower-field) regions. Because of this states with positive Stark shifts are often known as low-field-seeking states, while states with negative Stark shifts are known as high-field-seeking states. Forces of the kind described by Eq. (3), experienced by atoms or molecules possessing non-zero electric dipole moments in inhomogeneous electric fields, have played central roles in experiments involving focussing [7], state-selection [8–11], and ultimately multistage deceleration and trapping of polar ground state molecules [12–14], and are analogous to those exploited in the experiments of Gerlach and Stern involving silver atoms with non-zero magnetic dipole moments in inhomogeneous magnetic fields [15–17].

In addition to the large electric dipole moments that result from *ℓ*-mixing of hydrogenic Rydberg states in electric fields, the resulting Rydberg-Stark states also exhibit fluorescence lifetimes that are significantly longer than those of pure low- *ℓ* states typically prepared by laser photoexcitation. The fluorescence lifetimes of the Stark states with the largest electric dipole moments which can be prepared by two-photon excitation are on the order of 100 *μ*s for values of *n*>30 and scale with *n* ^3^ – *n* ^5^ [18].

Over that last 10 years experimental techniques have been developed to exploit these long lifetimes and large electric dipole moments to accelerate, decelerate, transport and trap gas-phase samples of atoms and molecules in hydrogenic Rydberg states using inhomogeneous electric fields. The ubiquity of hydrogenic Rydberg states in atoms and molecules means that these techniques, which are reviewed here, can be applied to a wide range of species including those that cannot be easily laser-cooled using current technologies [19, 20], homonuclear diatomic molecules which do not possess significant electric or magnetic dipole moments in their ground states and therefore cannot be readily decelerated using other methods, e.g., multistage Stark [12, 14] or Zeeman [21–23] deceleration, and exotic species such as the positronium atom with short-lived ground states that are prone to decay by annihilation [24, 25]. In this respect these Rydberg-Stark deceleration techniques represent one of several direct approaches to the preparation of chemically important cold molecules [26, 27], others include multistage Stark deceleration [12, 14], multistage Zeeman deceleration [21–23], optical Stark deceleration [28], buffer gas cooling [29, 30], and sympathetic cooling of molecular ions [31, 32].

In addition to the use of inhomogeneous electric fields, alternative approaches to trapping Rydberg atoms have also been developed. These include magnetic trapping Rb atoms in high-angular-momentum Rydberg states prepared by collisional *m*-mixing of low- *ℓ* Rydberg states with values of *n* close to 130 [33]. The investigation of approaches to magnetic trapping atoms in such high- *ℓ* states is of relevance to the production and confinement of anti-hydrogen [34]. In addition, confinement of Rb Rydberg atoms in optical lattices has also been achieved [35].

### Applications of decelerated beams of Rydberg atoms and molecules

Highly excited Rydberg states of atoms and molecules play important roles in many areas at the interface between physics and physical chemistry. Transitions between individual Rydberg states of carbon with values of *n* exceeding 1000 have been observed in absorption spectra following recombination in the interstellar medium [36]. In the upper atmosphere of the Earth atoms and molecules in excited states including Rydberg states, are also expected to play important roles in the decay processes and reactivity of atmospheric plasmas [37]. Rydberg-Stark deceleration and electric trapping of Rydberg atoms and molecules using the techniques reviewed here opens up opportunities to experimentally study these decay processes in controlled laboratory environments on timescales exceeding 1 ms, which, prior to the development of these methods, was not possible.

**Fig. 2:**
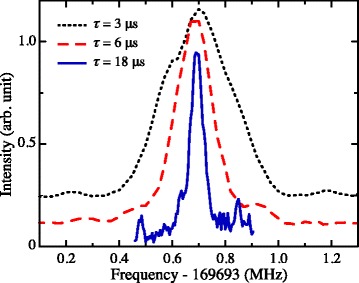
High-resolution millimeter-wave spectra of Kr. Millimeter-wave spectra of the 77d[3/2](*J* ^′^=1)→93p[3/2](*J*=1) transition in Kr recorded following interaction times between the atoms and the millimeter-wave field of *τ*=3, 6 and 18 *μ*s as indicated. The corresponding transition line-widths are 350, 180 and 60 kHz, respectively. From [6] with permission

High-resolution laser, millimeter-wave and microwave spectroscopy of atomic and molecular Rydberg states is of importance in studies of the role of nuclear spins in photoionisation [38], spectroscopic studies of the interactions of Rydberg atoms and molecules with surfaces [39, 40], and in the precise determination of ionisation and dissociation energies [41–44]. In many of these experiments the achievable frequency resolution is not limited by the bandwidth of the radiation sources used, but instead by the interaction times of the atomic or molecular samples with the radiation field. Examples of this effect of interaction-time broadening in vacuum-ultraviolet–millimeter-wave double-resonance spectra of the 77d[3/2](*J* ^′^=1)→93p[3/2](*J*=1) transition in Kr can be seen in Fig. 2 [6]. These spectra were recorded following preparation of the 77d[3/2](*J* ^′^=1) state by single-photon excitation from the ground state, after which the excited atoms interacted with a narrow-bandwidth millimeter-wave field for a period of time, *τ*, before the population in the 93p[3/2](*J*=1) state was detected by selective pulsed electric-field ionisation. The effect of interaction-time broadening can be clearly seen as *τ* is increased from 3 *μ*s to *τ*=18 *μ*s and the measured line-width decreases from 350 kHz to 60 kHz. The resolution in these experiments is not limited by the bandwidth of the millimeter-wave source but instead by the interaction time between the atoms and the radiation field. Further improvements in resolution in these experiments require longer interaction times. In precision spectroscopic studies of ground state atoms or molecules this is often achieved using the Ramsey method of separated oscillatory fields [45]. However, the sensitivity of high Rydberg states to stray or inhomogeneous electric fields makes it challenging to achieve sufficient control over these fields in an extended volume to exploit these methods. For this reason the most appropriate approach to increasing interaction times is to exploit decelerated beams.

The development of methods for preparing quantum-state-selected velocity-controlled beams of atoms and molecules which possess electric or magnetic dipole moments in their ground or low-lying metastable states has given rise to opportunities to perform low-energy scattering experiments with collision energy resolution on the order of *E*_kin_/*h* *c*=0.01 cm ^−1^ (see, e.g., [46, 47]). In a similar vein, a range of scattering studies, involving atoms or molecules in high Rydberg states, are expected to benefit from the opportunities to prepare cold, velocity-tuneable beams using Rydberg-Stark deceleration. These range from studies of the interactions of Rydberg atoms and molecules with surfaces, to investigations of energy transfer in collisions between samples of Rydberg atoms or molecules and ground states species.

The interactions of atoms and molecules in high Rydberg states with surfaces are of importance in several areas of research, including, e.g., cavity-quantum-electrodynamics at vacuum–solid-state interfaces [39, 48], experiments involving the photoexcitation of Rydberg states of samples confined in miniature vapor cells [49], and studies of charge transfer [3, 50]. At distances of <10 *μ*m from conducting surfaces, the interaction of a Rydberg atom or molecule with its image-dipole in the surface contributes to state-changing and attractive forces toward the surface [48, 51, 52]. Investigations of these processes are of importance in developing accurate models for charge-transfer (ionisation) into the surfaces [50, 53, 54]. Several studies of these surface-ionisation processes have been carried out using beams of Rydberg atoms and molecules. These have included experiments with beams of K and Xe atoms [50, 53] and H_2_ molecules [54], and with beams of H atoms prepared in Stark states with large electric dipole moments which permitted investigations of the role that the orientation of the electric dipole moment with respect to the surface had on the ionisation dynamics [55]. Recent experimental studies of resonant charge-transfer from H Rydberg atoms at Cu(100) surfaces have highlighted a dependence of the ionization process on the velocity of the incoming Rydberg atoms [56]. This would suggest that it will be of interest in future studies to exploit velocity-controlled or decelerated beams to obtain precise control over the positions and momenta of the atoms or molecules.

**Fig. 3:**
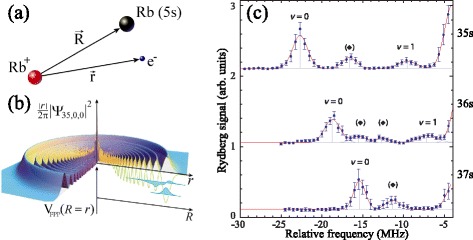
Long-range Rydberg molecules. **a** and (**b**) the scattering process involving an electron in a diffuse Rydberg orbital and a ground state Rb atom which gives rise to sets of bound molecular eigenstates. **c** Experimentally measured transitions to the lowest vibrational states of long-range Rb_2_ Rydberg molecules in the vicinity of the 37s (*bottom*), 36s (*middle*) and 35s (*top*) single-atom Rydberg states. From [67] with permission

The high polarizability and large electric dipole moments of high Rydberg states give rise to strong van der Waals, dipole-dipole and higher order electric multipole interactions [57, 58]. These have been exploited in laser cooled samples of ultracold atoms to blockade photoexcitation [59–61], study cooperative effects [62], and prepare multiparticle entangled states [63, 64]. However, these properties, which are a result of the large spatial extent of Rydberg electron wavefunctions, also play important roles in the interactions of samples in Rydberg states with ground state atoms or molecules [65]. Perhaps the most spectacular consequences of these interactions are seen in long-range Rydberg molecules [66, 67]. As depicted in Fig. 3(a) and (b) these molecules arise as a result of the scattering of a slow electron in a diffuse Rydberg orbital from a ground state atom or molecule. These bound molecular states have so far only been observed upon photoassociation in samples of laser cooled atoms [see Fig. 3(c)], however, it can be expected that they should also play a role in very-low–energy atomic or molecular scattering experiments in which one of the collision partners is prepared in a high Rydberg state. The development of methods such as those reviewed here for preparing a wider range of cold, decelerated beams of atoms and molecules in Rydberg states has the potential to open up exciting opportunities for studies of this unique gas-phase chemistry at long-range.

Highly excited Rydberg states of atoms and molecules are very sensitive to resonant electromagnetic fields at microwave or millimeter-wave frequencies. This is a consequence of (1) the fact that the energy differences between states for which *Δ* *n*=1 scale with *n* ^−3^ and correspond to transition frequencies <400 GHz for *n*≥25; and (2) the large electric dipole transition moments for these *Δ* *n*=1 transitions which scale with *n* ^2^ and approach ∼150 *e* *a*_0_ for *n*=25. These properties, combined with their long lifetimes, have seen Rydberg atoms play an important role in studies of microwave cavity quantum electrodynamics (QED) since the field was established [68].

Recent implementations of microwave cavity QED in two-dimensional surface-based superconducting microwave circuits [69] have led to a new role for Rydberg atoms in hybrid cavity QED experiments [70]. In these hybrid gas-phase–solid-state systems the Rydberg atoms are considered as long-coherence-time quantum bits (qubits) which will be coupled via two-dimensional chip-based superconducting microwave resonators to solid-state devices. These hybrid quantum systems take advantage of the long coherence times offered by gas-phase atoms as qubits, the strong coupling that can be achieved between Rydberg atoms and microwave resonators because of the their large electric dipole transition moments, and the scalability offered by micro-fabricated superconducting circuits to open new avenues of study in cavity QED at vacuum–solid-state interfaces, and potential applications in quantum information processing.

**Fig. 4:**
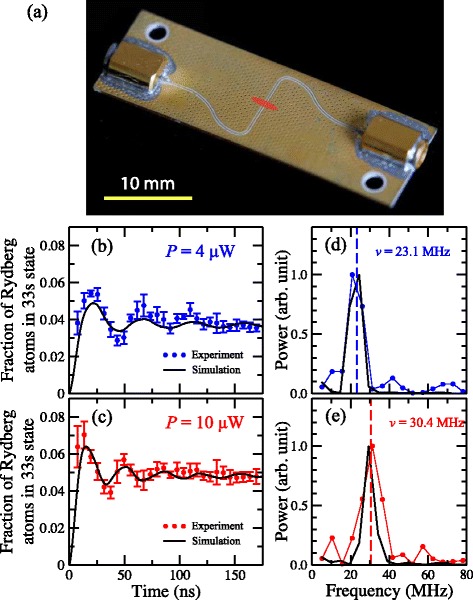
Coupling Rydberg atoms to microwave circuits. **a** Photograph of a coplanar microwave waveguide with the position of a sample of Rydberg atoms, when probed by a pulsed microwave field propagating along the transmission line, indicated schematically by the red shaded region. **b**-**c** Rabi oscillations observed in an ensemble of Rydberg atoms coupled to the microwave field surrounding the waveguide for microwave powers of 4 *μ*W and 10 *μ*W at the source. **d**-**e** Fourier transforms of (**b**) and (**c**). From [39]

Several approaches have been pursued in these experiments, including the realisation of atom-chips containing microwave circuitry with which cold samples of Rb have been prepared and then photoexcited to Rydberg states [71, 72], and the preparation of beams of Rydberg atoms which propagate above the surfaces containing the microwave circuits (see Fig. 4) [39]. The latter approach has several advantages. The chip-based circuits can be located in a cryogenic environment where direct laser access is not required, Rydberg state photoexcitation can be carried out in a region of the apparatus which is spatially separated from the microwave circuits permitting finer control over the initial-state preparation process, and atoms or molecules can be selected for use in the experiments to ensure that effects of adsorption on the cryogenic surfaces is minimised [40]. An example of the coherent coupling of beams of helium Rydberg atoms to pulsed microwave fields surrounding a co-planar waveguide are displayed in Fig. 4(b) and (c) [39]. The dependence of the observed Rabi frequency for microwave transitions between Rydberg states on the microwave power can be clearly seen in the Fourier transforms of the experimental and calculated data [Fig. 4(d) and (e)]. In these experiments the dephasing of the Rabi oscillations was dominated by the spatial spread of the Rydberg atom beam in the inhomogeneous stray electric fields above the surface of the waveguide and the motion of the atoms. This work has in part provided motivation for the development of the chip-based guides, decelerators and traps for beams of Rydberg atoms and molecules reviewed here.

Experimental techniques with which the translational motion of Rydberg atoms and molecules can be manipulated are of particular interest in experiments with antihydrogen ($\overline {\mathrm {H}}$) and positronium (Ps). Antihydrogen atoms formed by recombination of antiprotons and positrons [34], or by positron transfer in collisions between antiprotons and Rydberg positronium atoms [73–76] are produced in high Rydberg states. At present $\overline {\mathrm {H}}$ atoms that eventually decay to their ground state can be magnetically trapped [77, 78], however, no attempts have been made to confine the atoms while in the Rydberg states to improve the ultimate trapping efficiency after decay. In this respect, the implementation of methods to electrically trap Rydberg atoms in a wide rage of Stark states in the presence of strong background magnetic fields is of particular interest. In addition, the AEgIS experiment, currently under development at CERN, is designed to exploit inhomogeneous electric fields to accelerate Rydberg $\overline {\mathrm {H}}$ atoms to produce beams with tuneable velocities for antimatter gravity and spectroscopy experiments [76].

**Fig. 5:**
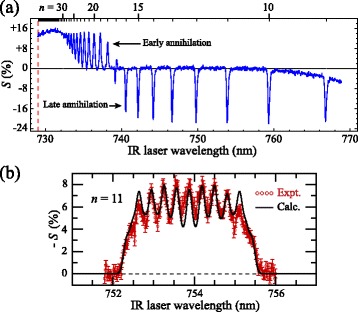
Spectra of Rydberg states of positronium. **a** Two-colour two-photon excitation spectrum of Rydberg states of Ps with values of *n* from 9 up to the ionisation limit. States with values of *n*>17 are detected when they annihilate after ionisation at a wire grid close to the photoexcitation region (*early annihilation*). States with values of *n*<17 pass through this grid and are detected when they annihilate at the walls of the vacuum chamber (*late annihilation*). **b** When photoexcitation is carried out in the presence of an electric field of 1.9 kV/cm individual Rydberg-Stark states can be selectively prepared. From [82]

The Ps atom, the bound state of an electron and a positron, is another unique system which is of interest in tests of the effect of the Earth’s gravitational field of particles composed of antimatter, [79, 80] and precision spectroscopy of fundamental importance [81]. The longer-lived triplet ground state of Ps has an annihilation lifetime of 142 ns [25], while the singlet state lives for 125 ps [24]. However, when excited to high Rydberg states, as in the data in Fig. 5, the spatial overlap of the electron and positron wavefunctions is reduced with the result that for all excited triplet levels other than the 2 ^3^S_1_ level the rate of fluorescence to the ground state is greater than the direct annihilation rate. Furthermore because of its reduced mass of exactly 0.5 *m*_e_, where *m*_e_ is the electron (≡ positron) mass, the fluorescence lifetimes of Rydberg states of Ps [82–85] are twice as long as those of states with the same values of *n* in the H atom. For this reason Rydberg states of Ps are well suited for precision spectroscopic studies, and tests of antimatter gravity. The challenge associated with carrying out precision spectroscopy or gravity measurements with Rydberg Ps is the high speeds (∼10^5^ m/s) with which the samples, produced by implantation of pulsed positron beams into room temperature porous silica targets, move [86]. However, these speeds correspond to kinetic energies *E*_kin_/*e*≃50 meV (*E*_kin_/*h* *c*≃460 cm ^−1^) that lie well within the ∼80 meV (≡650 cm ^−1^) changes in kinetic energy that have been achieved in Rydberg-Stark deceleration of fast beams of He Rydberg atoms using inhomogeneous electric fields [87]. As a result Rydberg-Stark deceleration represents a viable route to the preparation of slowly moving, or electrically trapped, Ps atoms.

## Rydberg states in electric fields

In the presence of an external electric field $\vec {F}=(0,0,F)$, the Hamilton, *H*, of an atom with a single excited Rydberg electron can be expressed as

4 $$\begin{array}{@{}rcl@{}} H &=& H_{0} + H_{\mathrm{S}}\\ &=& H_{0} + eFz, \end{array} $$

where *H*_0_ is the Hamiltonian in the absence of the field, *H*_S_ is the Stark Hamiltonian representing the interaction with the field, *e* is the electron charge, and *z* represents the position in cartesian coordinates.

### The Stark effect in Rydberg states of the hydrogen atom

**Fig. 6:**
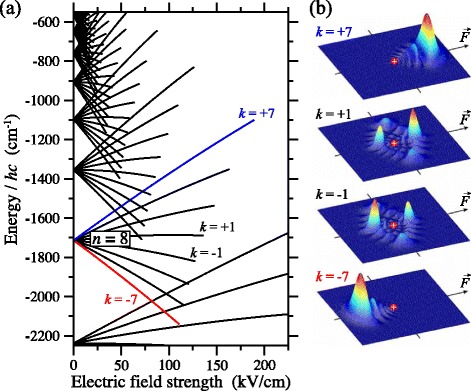
The Stark effect in Rydberg states of the H atom. **a** Dependence of the energies of *m*=0 Stark states with values of *n* from 7 to 14 on the strength of the electric field. **b** Electron probability density in a plane containing the electric field axis for *n*=8 Stark states with *k*=−7,−1,+1 and +7. After [90]

In hydrogenic Rydberg states the Schrödinger equation associated with the Hamiltonian *H* can be solved in parabolic coordinates [88, 89]. The wavefunctions obtained are characterised by four quantum numbers: *n*, *m* the azimuthal quantum number, and two parabolic quantum numbers *n*_1_ and *n*_2_. These quantum numbers satisfy the condition that *n*=*n*_1_+*n*_2_+|*m*|+1. The energies of the eigenstates, generally referred to as Stark states, in the presence of the field can be expressed to second order as [5]

5 $$\begin{array}{@{}rcl@{}} E_{n\,n_{1} n_{2} m\,} &=& E_{\text{ion}} -\frac{R_{M}\,hc}{n^{2}} + \frac{3}{2}n(n_{1}-n_{2})\,{ea}_{0}F +\\ & & -\frac{1}{16}n^{4}\left[17\,n^{2} - 3(n_{1}-n_{2})^{2} - 9\,m^{2} + 19\right]\frac{e^{2}{a_{0}^{2}}}{E_{\mathrm{h}}}F^{\,2} + \dots \end{array} $$

where *E*_h_=2*h* *c* *R*_*M*_ and *a*_0_ are the Hartree energy and the Bohr radius corrected for the reduced mass and the charge of the ion core, respectively. Often the difference between the two parabolic quantum numbers is denoted by the index *k*, such that *k*=*n*_1_−*n*_2_. For each vale of *m*, the allowed values of *k* range from −(*n*−|*m*|−1) to +(*n*−|*m*|−1) in intervals of 2. The resulting electric field dependence of the *m*=0 Stark states of the H atom with values of *n* ranging from 7 to 14 are displayed in Fig. 6(a). From this energy level diagram it can be seen that in weak electric fields the first order, linear Stark shift dominates with the quadratic and higher order terms gradually increasing in significance as the field strength increases. This effect is most visible in this figure for the states with values of *k* close to zero in this figure.

Comparison of the first order term in electric field strength in Eq. (5) with Eq. (2) indicates that to each Stark state an electric dipole moment of $\vec {\mu }_{n\,k}= (0, 0, -(3/2)nk\,e\,a_{0})$ can be attributed. These electric dipole moments are a consequence of the spatial distribution of electron charge about the ion core in each eigenstate, as is evident from the electron probability density distributions displayed in Fig. 6(b) for the *n*=8 Stark states with *k*=−7,−1,+1 and +7 [90]. The noticeable spatial separation of the positive charge of the ion core, from the distribution of negative charge associated with the electron, seen for the *k*=+7 Stark state in Fig. 6(b) indicates clearly that the electric dipole moment (214 D) of this state is oriented antiparallel to $\vec {F}$ giving rise to the positive Stark energy shift of this state in Fig. 6(a). In the same way the negative Stark shift of the *k*=−7 state results from the orientation of its electric dipole moment parallel to $\vec {F}$. As can be seen in Fig. 6(b) the states with low values of |*k*| in the middle of the Stark manifold have approximately equal distributions of electron charge on either side of the ion core and therefore small electric dipole moments (31 D) and weak linear Stark shifts. Typically Rydberg-Stark deceleration experiments have been performed following photoexcitation of outer Stark states with values of *n* between 15 and 60, and electric dipole moments between 500 D and 13500 D.

### Electric field ionisation of hydrogenic Rydberg states

In manipulating the translational motion of atoms or molecules in hydrogenic Rydberg-Stark states using inhomogeneous electric fields, the maximal fields that can be employed are limited by the ionisation electric fields of the states in which the samples are prepared. The Stark contribution, *H*_S_, to the Hamiltonian in Eq. (4) gives rise to a saddle point in the potential experienced by the Rydberg electron. If the energy of this saddle point lies below the energy of the excited Rydberg state, electric field ionisation will occur. This ionisation field depends strongly on the value of *n*, and also on the value of *k*. The outermost state with a negative Stark energy shift [e.g., the *k*=−7 state in Fig. 6(a)] typically ionises in fields equal to or larger than the classical ionisation field, *F*_class_, for which the energy of the Stark saddle point coincides with the energy of the Stark state in the field [18]

6 $$\begin{array}{@{}rcl@{}} F_{\text{class}} = \frac{F_{0}}{9n^{4}}, \end{array} $$

where *F*_0_=2*h* *c* *R*_M_/(*e* *a*_0_), with *R*_M_ and *a*_0_ adjusted to account for the reduced mass of the system and the charge of the ion core. More strictly the rate at which the electron tunnels through the barrier associated with the Stark saddle point must be considered in a complete description of the ionisation process. For a state |*n* *n*_1_ *n*_2_ *m*〉 the ionisation rate in an electric field *F* is [5]

7 $$\begin{array}{@{}rcl@{}} \Gamma_{n\,n_{1}\,n_{2}\,m} &=& \frac{E_{\mathrm{h}}}{\hbar}\frac{(4C)^{2n_{2}+m+1}}{n^{3}\,n_{2}!\,(n_{2}+m)!}\\ && \times\exp\left[-\frac{2}{3}C - \frac{1}{4}n^{3}\,\frac{e\,a_{0}\,F}{E_{\mathrm{h}}}\,\left(34{n_{2}^{2}}+34n_{2}m\phantom{\frac{53}{3}}\right.\right.\\ && \left.\left. +46n_{2}+7m^{2}+23m+\frac{53}{3} \right) \right] \end{array} $$

where

8 $$\begin{array}{@{}rcl@{}} C &=& \frac{1}{e\,a_{0}\sqrt{E_{\mathrm{h}}}}\frac{\left(-2E_{n\,n_{1}\,n_{2}\,m}\right)^{3/2}}{F} \end{array} $$

and $E_{n\,n_{1}\,n_{2}\,m}$ is the energy of the state, with respect to the field-free ionisation limit, in the presence of the electric field. With this in mind, the classical ionisation field, Eq. (6), corresponds to the field in which the ionisation rate of the *k*=−(*n*−1) state is ∼10^8^ s ^−1^. Typically electric field switching times on the order of 10 ns are achieved in experiments in which pulsed electric field ionisation is employed for the detection of Rydberg atoms or molecules. To ensure complete ionisation, fields that lead to ionisation rates on the order of 10^8^ s ^−1^ are therefore required. Because the Rydberg electron has a non-zero probability of being located on the side of the ion core opposite to the Stark saddle point for states with values of *k*>−(*n*−1), higher fields are required to achieve equivalent ionisation rates for these states. The result of this is that the fields for which similar ionisation rates occur for the *k*=+(*n*−1) states is approximately 2*F*_class_. Calculating the ionisation rate using Eq. (7), and accounting for energy shifts up to fourth-order in *F*, leads to ionisation rates of 10^8^ s ^−1^ for the *n*=30, *k*=−29 and *k*=+29 states of 740 V/cm and 1750 V/cm, respectively.

### Fluorescence lifetimes

**Fig. 7:**
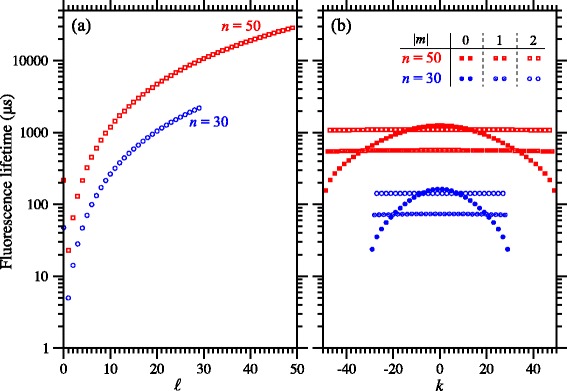
Fluorescence lifetimes of Rydberg states of the H atom. **a** Fluorescence lifetimes of field-free states of the H atom with *n*=30 and 50, for each allowed value of *ℓ*. **b** Fluorescence lifetimes of |*m*|=0,1 and 2 Rydberg-Stark states with *n*=30 and 50, and each allowed value of *k*

While an atom or molecule in a highly excited Rydberg state is energetically far from equilibrium, Rydberg-Stark states with sufficiently long fluorescence lifetimes to permit deceleration to zero mean velocity in the laboratory-fixed frame-of-reference and electric trapping can generally be photoexcited from a ground state, or low-lying intermediate state. The fluorescence rate, *Γ*_*nℓ*_, of an excited Rydberg state, |*n* *ℓ*〉, is given by the sum of the Einstein *A* coefficients associated with all allowed decay pathways to energetically lower-lying states, |*n* ^′^ *ℓ* ^′^〉, [88], i.e.,

9 $$\begin{array}{@{}rcl@{}} \Gamma_{n\ell} &=& \sum\limits_{n'\ell'} A_{n'\ell',n\ell}, \end{array} $$

where [18, 91]

10 $$\begin{array}{@{}rcl@{}} A_{n'\ell',n\ell} &=& \frac{2e^{2}\omega_{n'\ell',n\ell}^{3}}{3\epsilon_{0}\,h c^{3}}\frac{\ell_{\text{max}}}{2\ell+1} |\langle n'\ell'|r|n\ell\rangle|^{2}, \end{array} $$

with $\phantom {\dot {i}\!}\omega _{n'\ell ',n\ell }=2\pi \nu _{n'\ell ',n\ell }$ the angular frequency corresponding to the energy difference between the states, *ε*_0_ the vacuum permittivity, and *ℓ*_max_=max(*ℓ*,*ℓ* ^′^). The fluorescence lifetime, *τ*_*nℓ*_, of the excited state is then *τ*_*nℓ*_=(*Γ*_*nℓ*_)^−1^. Because of the dependence of the Einstein *A* coefficient on the cube of the transition frequency, the lifetimes of *n*p Rydberg states are typically dominated by decay to the ground state. For |*n* *ℓ*〉 Rydberg states with *ℓ*≠1, decay via a single-photon electric-dipole transition to the 1s cannot occur and, as can be seen in Fig. 7(a), longer lifetimes result.

The mixed- *ℓ* character of Rydberg-Stark states results in rates of fluorescence which lie between the fluorescence rates of the short-lived low- *ℓ* states and the longest-lived ‘circular’ *ℓ*=*n*−1 states. These fluorescence rates also have a strong dependence on the value of |*m*| since Stark states with higher values of |*m*| do not exhibit short-lived low- *ℓ* character because of the requirement that *ℓ*≥|*m*|. The fluorescence lifetime of each |*n* *n*_1_ *n*_2_ *m*〉 Rydberg-Stark state is determined by summing over the decay rates, *Γ*_*nℓ*_, associated with all allowed decay pathways from the |*n* *ℓ*〉 states into which it can be transformed weighted by the transformation coefficients [92]. These transformation coefficients can be expressed in terms of Wigner-3J symbols as [18],

11 $$\begin{array}{@{}rcl@{}} \langle {nn}_{1}n_{2}m|n\ell m\rangle &=& (-1)^{\left[(1-n+m+n_{1}-n_{2})/2\right] + \ell}\sqrt{2\ell+1}\\ & & \times \left(\begin{array}{ccc} \frac{n-1}{2} & \frac{n-1}{2} & \ell\\ \frac{m+n_{1}-n_{2}}{2} & \frac{m-n_{1}+n_{2}}{2} & -m \end{array}\right), \end{array} $$

such that

12 $$\begin{array}{@{}rcl@{}} |{nn}_{1}n_{2}m\rangle &=& \sum\limits_{\ell} |n\ell m\rangle\langle n\ell m|{nn}_{1}n_{2}m\rangle. \end{array} $$

Consequently the fluorescence rate, $\phantom {\dot {i}\!}\Gamma _{{nn}_{1}n_{2}m}$, of each individual Rydberg-Stark state is

13 $$\begin{array}{@{}rcl@{}} \Gamma_{{nn}_{1}n_{2}m} = \sum\limits_{n\ell} |\langle {nn}_{1}n_{2}m|n\ell m\rangle|^{2}\,\Gamma_{n\ell} \end{array} $$

and the fluorescence lifetime, $\tau _{{nn}_{1}n_{2}m}=\left (\Gamma _{{nn}_{1}n_{2}m}\right)^{-1}$. The fluorescence lifetimes of all *n*=30 and *n*=50 Rydberg-Stark states of the hydrogen atom for which |*m*|=0,1 and 2 are displayed in Fig. 7(b). From the data in this figure it can be seen that the fluorescence lifetimes of the *m*=0 Stark states exhibit a significant dependence on the value of *k*=*n*_1_−*n*_2_, while this is not the case for states with higher values of |*m*|. The fluorescence lifetimes of |*m*|=2 Stark states with values of *n*>30, which are typically prepared experimentally, exceed 140 *μ*s.

### The Stark effect in non-hydrogenic atoms and molecules

**Fig. 8:**
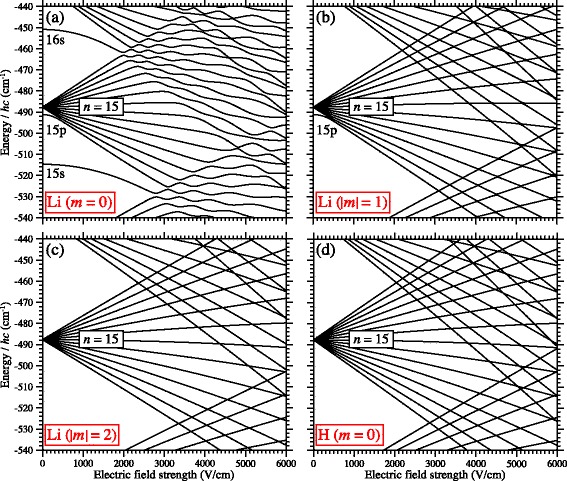
The Stark effect in non-hydrogenic atoms. Energy level diagrams depicting the Stark effect in *n*=15 Rydberg states of Li with **a** *m*=0, **b** |*m*|=1, and **c** |*m*|=2, and **d** *n*=15 Rydberg states of the H atom with *m*=0

In non-hydrogenic atoms and in molecules, the non-spherical symmetry of the ion core causes core-penetrating low- *ℓ* Rydberg states to be more strongly bound than the high- *ℓ* ‘hydrogenic’ states. This effect is accounted for in the Rydberg formula in Eq. (1) by the introduction of non-zero quantum defects, *δ*_*ℓ*_, for these low- *ℓ* states. In general the values of the quantum defects are most significant for states with *ℓ*≤4, while for higher- *ℓ* states *δ*_*ℓ*_≃0. In the presence of electric fields, non-hydrogenic low- *ℓ* Rydberg states exhibit quadratic Stark shifts in weak fields and give rise to large avoided crossings in Stark maps in higher fields. These effects can be seen clearly for the case of Li in Fig. 8(a). The quantum defects of the s-, p- and d-states close to *n*=15 used in these calculations were *δ*_s_=0.399, *δ*_p_=0.053 and *δ*_d_=0.002 [93]. Because of these non-zero quantum defects, the 15s and 15p states are shifted to lower energies than the higher- *ℓ* states. These states then exhibit quadratic Stark shifts in fields below ≈1000 V/cm and give rise to the large avoided crossings in fields beyond the Inglis-Teller limit, *F*_IT_=*F*_0_/(3*n* ^5^), where states with values of *n* which differ by 1 first overlap (e.g., for *n*=15, *F*_IT_≈2000 V/cm).

The calculation of the energy-level structure of non-hydrogenic Rydberg states of atoms and molecules in electric fields can be achieved by constructing the Hamiltonian matrix in a spherical |*n* *ℓ* *m*〉 basis and determining its eigenvalues. This approach has been employed previously to calculate the Stark effect in Rydberg states of alkali metal atoms [93]. In addition to being suited to treating non-hydrogenic atomic systems, this method has also been extended to Rydberg states of molecules [94–96]. The coefficients of the eigenvectors of the Hamiltonian matrix can then be employed to calculate the spectral intensities of transitions to, and the fluorescence lifetimes of, the resulting eigenstates.

The zero-field matrix is, to a good approximation, diagonal in the |*n* *ℓ* *m*〉 basis and the matrix elements can be calculated using Eq. (1). The term in the Hamiltonian in Eq. (4) representing the effect of the electric field is

14 $$\begin{array}{@{}rcl@{}} H_{\mathrm{S}} &=& e\,Fz\\ &=& e\,Fr\cos\theta, \end{array} $$

in spherical coordinates (*r*,*θ*,*ϕ*). This gives rise to matrix elements of the form [93]

15 $$\begin{array}{@{}rcl@{}} \langle n'\,\ell'\,m'|e\,Fr\cos\theta|n\,\ell\,m\rangle &=& e\,F\langle \ell'\,m'|\cos\theta|\ell\,m\rangle\langle n'\,\ell'|r|n\ell\rangle. \end{array} $$

Because

16 $$\begin{array}{@{}rcl@{}} \cos\theta = \sqrt{\frac{4\pi}{3}}\,Y_{1\,0}, \end{array} $$

the angular components of Eq. (15) can be determined by expansion in terms of spherical harmonics, *Y*_*ℓm*_. Therefore the angular matrix elements are zero unless *m* ^′^=*m* and *ℓ* ^′^=*ℓ*±1 (i.e., *Δ* *m*=0 and *Δ* *ℓ*=±1). Thus the external electric field mixes states with orbital angular momentum differing by one but does not give rise to *m*-mixing. Exploiting the properties of spherical harmonics [97] permits these non-zero angular integrals to be expressed analytically as [88, 93]

17 $$\begin{array}{@{}rcl@{}} \langle \ell+1\,m|\cos\theta|\ell\,m\rangle &=& \sqrt{\frac{(\ell+1)^{2} - m^{2}}{(2\ell+3)(2\ell+1)}} \end{array} $$

18 $$\begin{array}{@{}rcl@{}}\\ \langle \ell-1\,m|\cos\theta|\ell\,m\rangle &=& \sqrt{\frac{\ell^{2} - m^{2}}{(2\ell+1)(2\ell-1)}}. \end{array} $$

The radial matrix elements 〈*n* ^′^ *ℓ* ^′^|*r*|*n* *ℓ*〉 can be calculated analytically for the H atom [88]. For non-hydrogenic species, they can be determined numerically using the Numerov method [18, 93].

The precision of the Stark energy level structure calculated in this way depends on the accuracy of the quantum defects used and on the range of values of *n* included in the basis. Tests of convergence must be performed for the particular values of *n*, and the range of field strengths of interest. In weak fields, for which *F*≪*F*_IT_, contributions from matrix elements coupling states with different values of *n* are small and basis sets with only a small number of states are therefore often acceptable. However, in fields closer to, and beyond, the Inglis-Teller limit *n*-mixing plays a much more significant role with the result that larger basis sets are necessary.

Similar approaches can be employed in the calculation of the Stark effect in molecular Rydberg states. To account for the vibrational and rotational degrees of freedom of the molecular ion core the basis used must be extended. For example, in the calculation of the Stark effect in Rydberg states of H_2_, a Hund’s-case-(d) zero-field basis |*n* *ℓ* *N* ^+^ *N* *M*_*N*_>, where *N* ^+^ is the rotational angular momentum quantum number of the H$_{2}^{+}$ ion core, *N* is the total angular momentum quantum number excluding spin ($\vec {N}=\vec {N}^{+} + \vec {\ell }\,$), and *M*_*N*_ is the projection of $\vec {N}$ onto the electric field axis, is appropriate if vibrational channel interactions do not significantly perturb the spectra in the regions of interest [94, 98, 99].

Using this approach, and assuming that singlet-triplet mixing can be neglected, the diagonal elements of the electronic Hamiltonian matrix are first determined in a Hund’s-case-(b) basis |*n* *ℓ* *Λ* *N*>, where *Λ* is the quantum number associated with the projection of the electronic orbital angular momentum vector onto the internuclear axis. This facilitates the inclusion of the appropriate non-zero quantum defects, *δ*_*ℓΛ*_, for the low- *ℓ* Rydberg series converging to each rotational state of the ion core. In this basis, rotational channel interactions between series with a particular value of *ℓ* (i.e., for which *Δ* *ℓ*=0, while *ℓ*≤3) can then be included as off-diagonal matrix elements before transforming to the Hund’s-case-(d) basis to include the effects of the electric field. In this, Hund’s-case-(d) basis the energies of the rotational states of the ion core are then included as diagonal elements.

Applying the Stark Hamiltonian operator, Eq. (14), in the Hund’s-case-(d) basis leads to [94]

19 $$\begin{array}{@{}rcl@{}} \langle n'\ell' N'N^{+\prime}M_{N}'\left|eF z\right|n\ell NN^{+}M_{N}\rangle &=& eF\,\left(-1\right)^{N-M_{N}+N'+N^{+}+\ell+1}\\ &&\times\sqrt{\left(2N+1\right)\left(2N'+1\right)}\\ &&\times \left(\begin{array}{cll} N' & 1 & N\\ -M_{N'} & 0 & M_{N} \end{array} \right) \left\{ \begin{array}{cll} \ell' & N' & N^{+'}\\ N & \ell & 1\\ \end{array} \right\}\\ &&\times\langle n'\ell' |r|n\ell\rangle\,\delta_{N^{+}N^{+\prime}}\,, \end{array} $$

which has non-zero values only when *Δ* *ℓ*=±1, *Δ* *N*=0,±1 (0⇎0), and *Δ* *N* ^+^=0.

**Fig. 9:**
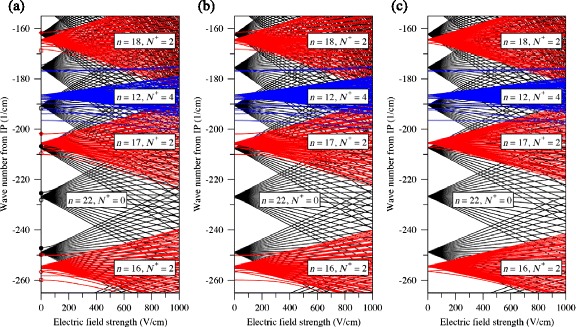
The Stark effect in Rydberg states of H_2_. Calculated Stark maps for para H_2_. *N* ^+^=0 states are indicated in black, *N* ^+^=2 states are indicated red, and *N* ^+^=4 states are indicated in blue. **a** |*M*_*J*_|=0, **b** |*M*_*J*_|=1, and **c** |*M*_*J*_|=3. From [98]

Stark energy level diagrams, calculated following this procedure for |*M*_*N*_|≡|*M*_*J*_|=0,1 and 2 Rydberg states of para-H_2_, from −265 cm ^−1^ to −155 cm ^−1^ with respect to the adiabatic ionisation limit are displayed in Fig. 9. This wavenumber range encompasses *N* ^+^=0 states for which *n*=21→27, *N* ^+^=2 states for which *n*=16→18, and the *N* ^+^=4 states for which *n*=12 [98].

### Lifetimes of molecular Rydberg states

In general, the fluorescences lifetimes of Rydberg states of molecules are similar to those in atoms, i.e., the fluorescence lifetimes of the pure low- *ℓ* states exceed 1 *μ*s for values of *n*>30, while those of the shortest-lived Rydberg-Stark states are 10 – 100 times longer. However, molecular Rydberg states can also decay by predissociation. This involves the transfer of energy from the Rydberg electron to the nuclear degrees of freedom of the molecule, typically as a result of the interaction between the potential energy surface of the Rydberg state with that of a repulsive valence state. The outcome of this process is the decay of the molecule into two or more fragments in their ground or excited states [2, 3].

Most rapid predissociation occurs on timescales ≪1 *μ*s. As a result, in general predissociative states are not well suited to Rydberg-Stark deceleration for which lifetimes $\gtrsim 10~\mu $s are required. However, the molecular Rydberg states which exhibit the most rapid predissociation are those of core-penetrating low- *ℓ* character, for which the Rydberg electron has a significant charge density in the vicinity of the ion core. For Rydberg electrons in higher *ℓ* states, the centrifugal barrier ensures that they do not significantly perturb the bond in the molecular ion core and predissociation can be inhibited. Because the quantum defects of high Rydberg states depend directly on the penetration of the Rydberg electron into the ion core, molecular Rydberg states with large quantum defects tend to be significantly more susceptible to predissociation than states with quantum defects approaching zero.

### Hydrogenic Rydberg-Stark states of non-hydrogenic atoms and molecules

For greatest efficiency, Rydberg-Stark deceleration should be implemented using hydrogenic Rydberg-Stark states with linear Stark energy shifts and lifetimes exceeding ∼10 *μ*s. Ideally the only limitation imposed on the electric fields employed should be that they remain at all times below the ionisation field. In hydrogenic atoms this condition is readily met. However, in non-hydrogenic species the avoided crossings at and beyond the Inglis-Teller limit [see Figs. 8(a) and 9(a)] are often too large to be traversed diabatically during deceleration. The result of this is that for *F*>*F*_IT_ the Stark states lose their electric dipole moments and cannot be efficiently manipulated in these fields. In molecules, predissociation of low- *ℓ* Rydberg states also results in lifetimes which are insufficient for deceleration.

However, these two challenges associated with decelerating non-hydrogenic atoms and molecules can be circumvented simultaneously by preparing states which do not have core-penetrating low- *ℓ* character. This is achieved by exploiting the selection rules for electric dipole transitions in the photoexcitation process to control the absolute value of the azimuthal quantum number, |*m*|, of the Rydberg states. By carefully controlling the value of |*m*|, *ℓ*-mixing induced by the electric fields can be restricted to states for which *ℓ*≥|*m*|. For example, if Rydberg states with |*m*|=1 are prepared in Li, Fig. 8(b), the Stark states do not possess s-character. As a result, the avoided crossings at and beyond the Inglis-Teller limit are reduced (from ∼1.4 cm ^−1^≡42 GHz to ∼0.1 cm ^−1^≡3 GHz). Preparation of states with |*m*|=2, Fig. 8(c), removes the contribution from the p-states, the other states with a significant non-zero quantum defect, resulting in a Stark map which is almost identical to that of the hydrogen atom with *m*=0, Fig. 8(d). The primary difference between the Stark maps in Fig. 8(c) and (d) is that in the former the outermost Stark states are absent. Photoexcitation of |*m*|=2 Rydberg-Stark states can readily be achieved in Li using a resonance-enhanced two-color two-photon excitation scheme from the 1s^2^2s ^2^S _1/2_ ground state driven using circularly polarised laser radiation with the same helicity for both steps of the excitation process, and propagating parallel to the electric field in the photoexcitation region. Alternatively, non-resonance enhanced single-color two-photon excitation using circularly polarised radiation could also be employed [100].

Multiphoton excitation schemes can also be implemented in molecules for the preparation of long-lived hydrogenic Rydberg states. The dependence of the Stark maps of para-H_2_ on the value of |*M*_*J*_| can be seen in Fig. 9. In this case, if photoexcitation to non–core-penetrating Rydberg states with *ℓ*≥3 is carried out, predissociation is inhibited and the resulting long-lived Rydberg-Stark states with *N* ^+^=0 exhibit a hydrogenic behaviour. These states have been prepared in deceleration and trapping experiments by resonance-enhanced three-color three-photon excitation using circularly polarised laser radiation following the excitation scheme [98, 101]

20 $$\begin{array}{@{}rcl@{}} &&\left[(1\mathrm{s}\,\sigma_{\mathrm{g}})^{1}(v^{+}=0,N^{+}=0)\right]\, (n\mathrm{f})^{1},(v=0, J=3,|M_{J}|=3)\\ &&\stackrel{\:\,\circlearrowleft}{\longleftarrow}\,(1\mathrm{s}\,\sigma_{\mathrm{g}})^{1}\, (3\mathrm{d}\,\pi_{\mathrm g})^{1}\,\mathrm{I}^{1}\Pi_{\mathrm g}(v'=0,J'=2,|M_{J}'|=2)\\ &&\stackrel{\:\,\circlearrowleft}{\longleftarrow}\,(1\mathrm{s}\,\sigma_{\mathrm{g}})^{1}\, (2\mathrm{p}\,\sigma_{\mathrm u})^{1}\,\mathrm{B}^{1}\Sigma^{+}_{\mathrm u}(v^{\prime\prime}=3,J^{\prime\prime}=1,|M_{J}^{\prime\prime}|=1)\\ &&\stackrel{\:\,\circlearrowleft}{\longleftarrow}\,(1\mathrm{s}\,\sigma_{\mathrm{g}})^{2}\,\mathrm{X}^{1}\Sigma^{+}_{\mathrm g}(v^{\prime\prime\prime}=0,J^{\prime\prime\prime}=0,|M_{J}^{\prime\prime\prime}|=0), \end{array} $$

which raises, after each step, the value of *ℓ*, *J* and |*M*_*J*_| by one. This approach to the preparation of long-lived molecular Rydberg states using carefully chosen multiphoton excitation schemes is quite general and could also be applied to other molecules. Decelerated and trapped molecular samples in hydrogenic Rydberg-Stark states offer the opportunity to observe slow predissociation processes and study their sensitivity to external fields, blackbody radiation and collisions on timescales that are very difficult to achieve in traditional beam experiments. To study collisions and decay processes in states with low- *ℓ* character microwave transitions could be exploited to efficiently change the value of |*M*_*J*_| after deceleration and trapping.

### Effects of blackbody radiation on high Rydberg states

**Fig. 10:**
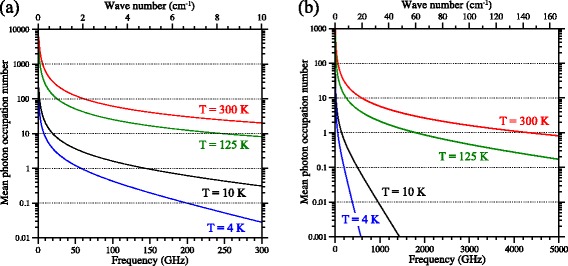
Thermal photon occupation numbers. Mean blackbody photon occupation number (**a**) at frequencies up to 300 GHz (10 cm ^−1^) and (**b**) at frequencies up to 5000 GHz (160 cm ^−1^), for blackbody temperatures of 300 K, 125 K, 10 K and 4 K

The small *Δ* *n*=1 energy intervals and large electric dipole transition moments of Rydberg states of atoms and molecules make them very sensitive to low-frequency (microwave or millimeter-wave) electromagnetic radiation. In particular, the blackbody radiation field of the environment surrounding the Rydberg atoms or molecules can have a significant effect on the Rydberg state population giving rise to energy level shifts [102], transitions between Rydberg states [103, 104] and photoionisation [105, 106]. The most important aspect of the blackbody radiation field in the treatment of its interaction with a Rydberg atom or molecule is the mean photon occupation number per mode $\overline {n}(\nu)$. $\overline {n}(\nu)$ represents the average number of blackbody photons of one polarisation, with frequency *ν*, at a temperature *T*, and can be expressed as [107]

21 $$\begin{array}{@{}rcl@{}} \overline{n}(\nu) &=& \frac{1}{\mathrm{e}^{h\nu/k_{\mathrm{B}}T}-1}, \end{array} $$

where *k*_B_ is the Boltzmann constant. Mean photon occupation numbers for blackbody temperatures of 300 K, 125 K, 10 K and 4 K are presented in Fig. 10(a) and (b) over two different frequency and wavenumber ranges.

In such a thermal radiation field, the transition rate from an initial state |*n* *ℓ* *m*〉 to a state |*n* ^′^ *ℓ* ^′^ *m* ^′^〉, is given by the product of the mean photon occupation number at the frequency of the transition, and the Einstein A coefficient for the transition. For example, at *n*=30 in the H atom, the wavenumber of the transition from the 30s state to the 31p state is 7.74 cm ^−1^ (≡232 GHz) and the radial integral |〈31p|*r*|30s〉|=316 *a*_0_. At room temperature (300 K), the mean photon occupation number at this frequency is $\overline {n}(\nu =232~\text {GHz})=26$, and therefore the blackbody transition rate is 2 436 s ^−1^. After summing the transition rates to all neighbouring Rydberg states, the total blackbody depopulation rate for H atoms in the absence of external fields at *n*=30 can be determined to be ∼12 400 s ^−1^ which corresponds to a time constant of ∼80 *μ*s.

In the presence of an electric field, the rates of *n*-changing transitions driven by blackbody radiation are highest between states with electric dipole moments with a similar orientation with respect to the electric field, and of similar magnitude. Consequently, for an atom or molecule in an outer Rydberg-Stark state with a positive Stark shift confined in an electrostatic trap, the blackbody transitions with the highest rate will be *Δ* *n*=±1 transitions to other trapped states which also exhibit positive Stark shifts. As a result, *n*-changing transitions driven by blackbody radiation do not in general lead to an immediate loss of atoms or molecules from an electrostatic trap. However, direct blackbody photoionisation of trapped atoms or molecules does lead to trap loss and plays a significant role on timescales on the order of 100 *μ*s for states with values of *n* close to 30 in a room temperature environment [105].

## Rydberg-Stark deceleration

The first proposals to exploit the large electric dipole moments associated with highly excited Rydberg states for deceleration and electrostatic trapping of atoms and molecules were advanced in the early 1980s. In an article on “*Electrostatic trapping of neutral atomic particles*” in 1980, Wing pointed out that “*At moderate field strengths Rydberg atoms have trap depths comparable to ambient kT...*” [108]. While in their article on “*Stark acceleration of Rydberg atoms in inhomogeneous electric fields*” in 1981, Breeden and Metcalf wrote that “*Rydberg atoms exhibit large electric dipole moments suggesting that inhomogeneous fields can exert forces on them*” and that “*... the resultant change in kinetic energy is equal to the Stark shift of the Rydberg state*” [109].

### Deceleration in time-independent electric fields

**Fig. 11:**
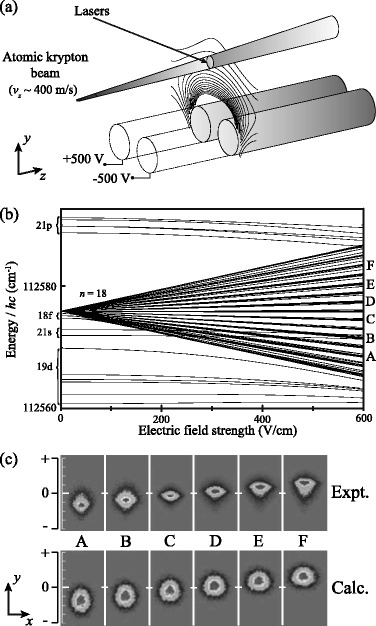
Transverse deflection of beams of Kr Rydberg atoms. **a** Schematic diagram of the experimental setup, including the inhomogeneous dipolar electric field distribution above the two cylindrical metallic rods, used to transversely deflect beams of Kr atoms. **b** Stark map for Rydberg states with values of *n* close to 18 in Kr. The Stark states labelled A to F on the righthand side of the figure were selectively excited and subjected to the deflection fields. **c** Experimentally recorded (Expt.) and calculated (Calc.) images of beams of atoms after deflection. From [110] with permission

First experiments in which the interactions of samples in Rydberg states with inhomogeneous electric fields were studied were reported in 2001 by Softley and co-workers [110]. This work involved the deflection of a pulsed supersonic beam of Kr atoms in the time-independent dipolar electric-field distribution surrounding a pair of cylindrical electrodes as depicted in Fig. 11(a). By selectively photoexciting Rydberg-Stark states, each with a different electric dipole moment [see Fig. 11(b)], directly above the pair of electrodes, deflection in the *y*-dimension, toward or away from the dipole was observed depending on the orientation and magnitude of the dipole moments of the states. This deflection was monitored by imaging the Rydberg atoms when they impinged upon a microchannel plate (MCP) detector Fig. 11(c).

**Fig. 12:**
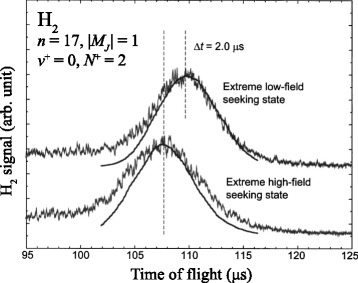
Acceleration and deceleration of beams of H_2_. Time-of-flight distributions of H_2_ molecules in extreme outer low-field-seeking (*upper trace*) and high-field-seeking (*lower trace*) Rydberg-Stark states for which *n*=17, after exiting a time-independent inhomogeneous electric field. From [99] with permission

These first experiments subsequently led to a proof-of-principle demonstration of the longitudinal acceleration of fast beams of hydrogen molecules in time-independent fields [99, 111] (see Fig. 12). In this work, a difference in the time of flight of the molecules over a fixed distance was observed for beams of H_2_ in the extreme low-field-seeking and extreme high-field-seeking *n*=17 Stark states of the *ν* ^+^=0, *N* ^+^=2 Rydberg series when the molecules were decelerated or accelerated in the inhomogeneous electric field of a pair of cylindrical electrodes, respectively.

Following this, experiments were carried out by Vliegen, Merkt and co-workers [112] using pulsed supersonic beams of Ar atoms. These studies were first performed in the static inhomogeneous electric field distribution of a pair of electrodes in a wedge configuration, generating an electric field gradient along the axis of the atomic beam. The effects of non-hydrogenic low- *ℓ* Rydberg states on the deceleration process in electric fields at and beyond the Inglis-Teller limit were identified in this work. The observation was made that the avoided crossings in these regions of the Stark map were traversed adiabatically under the conditions of the experiments. However, for Rydberg-Stark states of the H atom the opposite behavior was seen, with energy level crossings in fields beyond the Inglis-Teller limit traversed diabatically [113]. This confirmed that the Runge-Lenz vector remains conserved for hydrogenic systems in these deceleration experiments [89].

### Deceleration in time-dependent electric fields

**Fig. 13:**
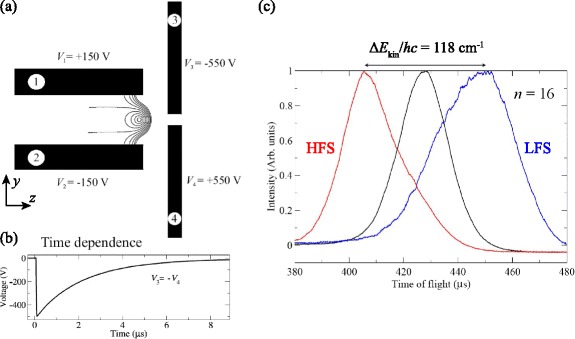
Acceleration and deceleration of beams of Ar atoms in time-dependent electric fields. **a** Electrode configuration used in the acceleration/deceleration of Ar Rydberg atoms using time-dependent inhomogeneous electric fields. **b** Time dependence of the potentials applied to electrodes 3 and 4 in (**a**) for acceleration/deceleration. **c** Experimentally recorded time-of-flight distributions demonstrating the acceleration (*left-hand red dataset*), deceleration (*right-hand blue dataset*) of high-field-seeking (HFS) and low-field-seeking (LFS) *n*=16 Rydberg-Stark states, respectively. The central black dataset represents the time-of-flight distribution of the undecelerated Rydberg atom beam. From [114] with permission

By introducing time-dependent electric fields the efficiency of the deceleration process could be significantly enhanced, particularly for non-hydrogenic species [114]. Using time-dependent potentials, large, continuously moving, electric field gradients could be generated at the position of the accelerating or decelerating Rydberg atoms while ensuring that the strength of the field was maintained below the Inglis-Teller limit where non-hydrogenic contributions to the deceleration process are most significant [112]. Applying potentials which exponentially decayed in time to a set of four electrodes in a quadrupole configuration, Fig. 13(a–b), permitted the mean longitudinal kinetic energy of beams of Ar atoms to be modified by up to *Δ* *E*_kin_/*h* *c*∼±60 cm ^−1^ [see Fig. 13(c)]. This change in kinetic energy is 2.7 times the Stark energy in the maximal field experienced by the atoms during acceleration/deceleration. In addition to ensuring that non-hydrogenic samples are not subjected to fields larger than the Inglis-Teller field during deceleration, time-dependent fields can also be exploited to maximise the efficiency with which H atoms, or other atoms or molecules in hydrogenic high- |*m*| states, can be decelerated while ensuring that they do not experience fields that could result in ionisation during deceleration.

### Rydberg atom mirror

**Fig. 14:**
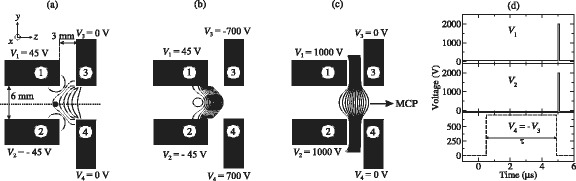
Electrode configuration of a Rydberg atom mirror. **a**–**c** Schematic diagrams of the set of metallic electrodes used to reflect beams of H Rydberg atoms in a normal incidence mirror. The electric potentials and corresponding field distributions at the time of (**a**) photoexcitation, (**b**) deceleration/reflection, and (**c**) detection are displayed. **c** Time-dependence of the electric potentials applied to each mirror electrode. From [115] with permission

The use of time-dependent electric fields subsequently permitted the demonstration of transverse focussing of beams of Ar atoms [115] and the reflection of beams of H atoms in a normal incidence Rydberg atom mirror [116]. The arrangement of metallic electrodes used to realise this mirror are depicted schematically in Fig. 14. In this figure the beam of ground state H atoms propagates in the *z*-dimension between the four mirror electrodes. At the time of photoexcitation, Fig. 14(a), a sufficiently homogeneous electric field was generated at the position between electrodes 1 and 2 where the lasers used for Rydberg state photoexcitation crossed the atomic beam (shaded circle between electrodes 1 and 2) to permit selective excitation of individual |*n*,*k*〉=|27,18〉 Rydberg-Stark states. After photoexcitation, pulsed potentials of ±700 V were rapidly applied to electrodes 3 and 4 resulting in a large positive electric field gradient at the position of the excited atoms. This gradient caused atoms in low-field-seeking Rydberg-Stark states to decelerate and was large enough that, if it persisted for a sufficient period of time, the atoms initially travelling at 720 m/s could be decelerated to a standstill and reflected into the negative *z*-dimension. In these experiments, Rydberg atoms located in the region between the four electrodes were detected by pulsed electric field ionisation. This was achieved by applying pulsed potentials of +1000 V to electrodes 1 and 2 simultaneously [see Fig. 14(c) and (d)] generating a large field to ionise the excited atoms and accelerate the resulting ions toward a MCP detector located further downstream in the apparatus.

**Fig. 15:**
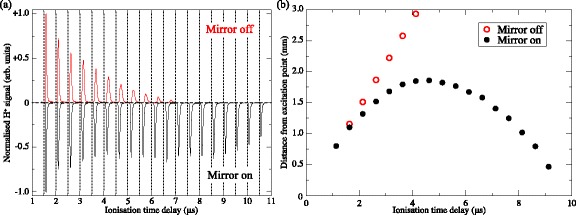
Measurements of reflected H Rydberg atoms. **a** Individual H ^+^ time of flight distributions recorded after pulsed electric field ionisation of atoms located between electrodes 1 to 4 in Fig. 14(a) at the times indicated by the dashed vertical lines. Positive-going datasets were recorded with the mirror off, while the negative-going, inverted datasets, were recorded with the mirror on. **b** Dependence of the mean longitudinal position of the cloud of Rydberg atoms, with respect to the position of photoexcitation, on the time delay before pulsed electric field ionisation as extracted from the data in (**a**) with the mirror off (*open red circles*), and on (*filled black circles*). From [116] and [150] with permission

The operation of this Rydberg atom mirror can be most directly seen by comparing the Rydberg atom ionisation signals with the mirror off, and with it active. When off, the Rydberg atoms fly through the region between the four electrodes where they can be ionised by the pulsed electric field, Fig. 14(c), within approximately 6 *μ*s of photoexcitation [Fig. 15(a) positive-going time-of-flight distributions]. On the other hand, if the mirror potentials are activated to decelerated the atoms the H ^+^ ion signal persist for more than 10 *μ*s [Fig. 15(a) inverted negative-going time-of-flight distributions]. This indicates that the electric field gradient associated with the Rydberg atom mirror decelerates the atoms sufficiently that they remain within the detection region for this longer period of time.

However, more detailed information on the longitudinal position of the Rydberg atoms at each ionisation time can be extracted from the ion time-of-flight distributions. In the case of the measurements with the mirror off, it can be seen in Fig. 15(a) that the flight-time of the H ^+^ ions to the MCP detector (i.e., the time interval between each dashed vertical line and the subsequent maximum in the time-of-flight distribution) increases as the delay between excitation and pulsed electric field ionisation increases. This occurs because when the Rydberg atoms move further into the positive *z* dimension within the mirror electrodes, the H ^+^ ions produced by pulsed electric field ionisation are accelerated through a smaller electric potential difference and therefore travel more slowly to the MCP. If the relation between the flight-time of the ions to the MCP and their position of ionisation is calibrated using a beam with a known longitudinal speed, the position of the atoms at the time of ionisation can be determined. With the mirror off, these positions are indicated by the open red circles in Fig. 15(b).

With this in mind, it can be seen in the inverted negative-going dataset in Fig. 15(a) recorded with the mirror activated, that although the flight-times of the ions to the MCP gradually increase for early ionisation times, at later times they reduce again. This behaviour is indicative of the atoms first moving forward into the position *z*-dimension and then being reflected backwards. Making the appropriate conversion from the mean time-of-flight of the H ^+^ ions, to the position of ionisation, the trajectory of the ensemble of Rydberg atoms in the *z*-dimension could be reconstructed [Fig. 15(b) filled black circles]. This shows that the atoms were brought from their initial longitudinal speed of 720 m/s, to a standstill ∼1.75 mm from their position of photoexcitation in a time of ∼4.5 *μ*s. In this process they experience an average acceleration of ∼−1.5×10^8^ m/s^2^.

## Electrostatic trapping Rydberg atoms and molecules

Using time-dependent electric potentials permits deceleration of Rydberg atoms or molecules in a continuously moving electric field gradient. In the electrode configuration used in the experiments described above these gradients form one side of a travelling electric quadrupole trap [see e.g., Fig. 13(a)]. Such a trap is suitable for confining atoms or molecules in low-field-seeking Rydberg-Stark states. Therefore, if sufficient kinetic energy is removed in the deceleration process and quadrupole electric field distributions are generated with minima at the positions of the decelerated samples, electrostatic trapping can be achieved using only a single deceleration stage [117]. This is the operation principle upon which a set of on-axis and off-axis three-dimensional electrostatic traps for Rydberg atoms and molecules have been developed.

### Trapping hydrogen and deuterium atoms

#### On-axis trap

The first three-dimensional electrostatic trap for atoms in selected Rydberg-Stark states was designed to act as a single-stage decelerator and a trap in which non-zero electric field minima could be generated [118]. The field gradients around this minimum gave rise to forces that confined atoms or molecules in low-field-seeking states. The electric potentials used in the first implementation of this device were optimised for H atoms in states for which |*n*,*k*〉=|30,25〉. These states possess electric dipole moments of ∼2900 D.

**Fig. 16:**
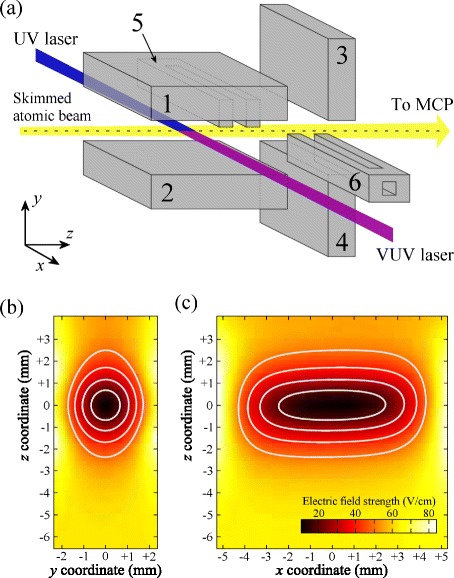
Three-dimensional electrostatic trap. **a** Schematic diagram of a single-stage Rydberg-Stark decelerator and three-dimensional electrostatic trap. **b** and (**c**) electric field distributions in the *yz* and *xz* planes at the center of the trap with potentials of |*V*_1,2,3,4_|=20 V and |*V*_5,6_|=55 V. The contour lines are spaced by 10 V/cm with the center-most corresponding to a field of 20 V/cm. The color bar indicating the field strength in (**c**) also holds for (**b**). From [118]

In these experiments, pulsed supersonic beams of H atoms with a mean longitudinal velocity of 665 m/s were generated by photolysis of NH_3_ seeded in Ar [119]. After entering the electrode arrangement presented in Fig. 16(a) the atoms were photoexcited to high Rydberg states using a resonance-enhanced two-colour two-photon excitation scheme via the 2 ^2^ *P*_1/2_ level. The operation of the trap required the application of potentials of +20 V (−20 V) to electrodes 1 and 4 (electrodes 2 and 3) to form a quadrupole electric field distribution in the *yz* plane with its minimum located at the mid-point between the four electrodes as in Fig. 16(b). To close off the trap in the *x* dimension, and set the minimum electric field to ∼9 V/cm, electrodes 5 and 6 were operated at +55 V and −55 V, respectively [see Fig. 16(c)]. This ensured that a quantisation axis was maintained throughout the trap volume and atoms would not be lost from the trap through non-adiabatic transitions to untrapped high-field-seeking states. In this configuration the trap had a depth of *E*/*h* *c*=2.2 cm ^−1^ (or *E*/*k*_B_= 3.2 K) for atoms in |*n*,*k*〉=|30,25〉 states.

**Fig. 17:**
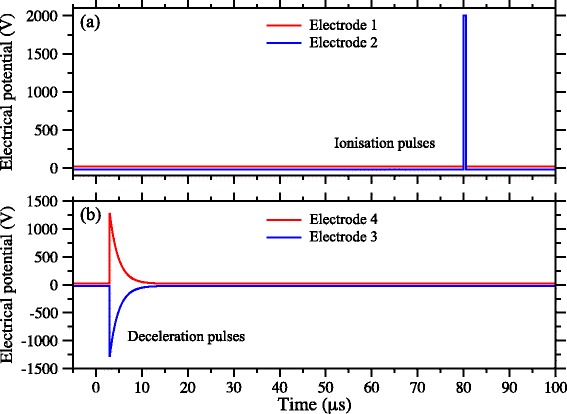
Time-dependent deceleration and trapping potentials. Time dependence of the electric potentials applied to electrodes 1–4 in Fig. 16(a). **a** Ionisation pulses applied to electrodes 1 and 2 to detect the trapped Rydberg atoms. **b** Exponentially decaying deceleration potentials applied to electrodes 3 and 4. The horizontal axis represents the time after photoexcitation

**Fig. 18:**
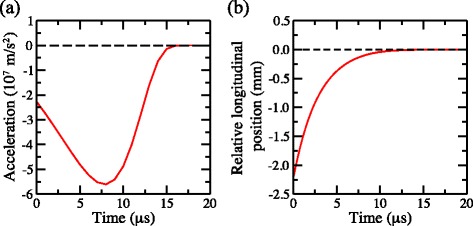
Acceleration and relative position of atoms during deceleration and trap loading. **a** Acceleration, and (**b**) relative longitudinal position in the *z* dimension with respect to the final position of the trap minimum, of H atoms in the |*n*,*k*〉=|30,25〉 state for which the time-dependence of the deceleration potentials was optimised. The origin of the horizontal axis is the activation time of the deceleration potentials

To decelerate the Rydberg atoms and load them into this trap, shortly after photoexcitation pulsed potentials of ±1265 V were applied to electrodes 3 and 4 (see Fig. 17). This gave rise to a large positive electric-field gradient along the *z* axis in which atoms in low-field-seeking states were decelerated. After being rapidly switched on, these potentials decayed exponentially with a time constant of 1.9 *μ*s. This exponential decay was optimised so that the decelerating atoms were always subjected to the maximum electric-field gradient that could be generated in the decelerator while never experiencing electric fields large enough to ionise them. The time dependence of the acceleration experienced by the atoms during the deceleration process was determined in numerical calculations of particle trajectories in the decelerator and are presented in Fig. 18(a). The origin of the horizontal axis in this figure represents the time at which the deceleration potentials applied to electrodes 3 and 4 were switched on. The largest acceleration experienced by the |*n*,*k*〉=|30,25〉 H Rydberg atoms was −5.5×10^7^ m/s^2^ and they were decelerated to zero velocity within ∼10 *μ*s of the initial rise of the deceleration potentials, after travelling ∼2.0 mm in the *z* dimension [see Fig.18(b)]. At the end of the deceleration process, the potentials applied to electrodes 3 and 4 returned to their initial values of ±20 V [see Fig. 17(b)]. Two unique and essential aspects of this deceleration and trapping procedure are (1) that the Rydberg-atom cloud is stopped exactly at the minimum of the trap with no transverse loss of atoms in the final stages of trap loading, and (2) that the atoms never traverse regions of zero electric field and therefore do not undergo randomisation of *k* and *m*.

The presence of Rydberg atoms within the trap volume was detected by pulsed electric-field ionisation using potentials of +2 kV with rise times of 50 ns and durations of ∼100 ns applied to electrodes 1 and 2 [see Fig.17(a)]. The resulting H ^+^ ions were then accelerated toward a MCP detector with phosphor screen positioned 20 cm from the trap minimum along the *z* axis.

**Fig. 19:**
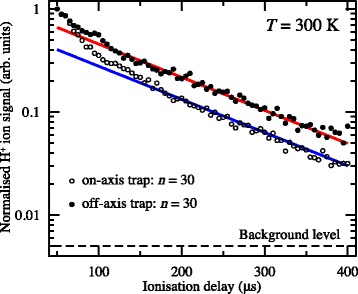
Decay of H Rydberg atoms from on-axis and off-axis electrostatic traps. On-axis (*open circles*) and off-axis (*filled circles*) decay of H atoms initially prepared in *n*=30 Rydberg-Stark states from traps operated at 300 K. The solid lines are single-exponential functions fitted to the experimental data beyond 200 *μ*s. From [105]

The integrated H ^+^ signal recorded as a function of the time delay between photoexcitation and field ionisation in this on-axis electrostatic trap is presented in Fig. 19 (open circles). Initially, the atom number density in the trap is 10^6^−10^7^ cm ^−3^. Fitting a single exponential function to the data beyond 200 *μ*s in this figure reveals two time periods in which the trap loss rates are quite different. At early times, up to ∼150 *μ*s, a rapid loss of atoms from the trap occurs which was seen to be strongly dependent on the density of the atomic beam [117]. This loss of atoms at early trapping times was attributed to collisions with the trailing components of the gas pulse. Beyond 200 *μ*s the exponential function fitted to the experimental data has a time constant of 135 *μ*s. This rate of decay of atoms from the trap results from a combination of the fluorescence lifetime of the Rydberg states prepared at photoexcitation, the rate at which transitions driven by the local room-temperature blackbody radiation field occur, and interactions between the trapped atoms.

**Fig. 20:**
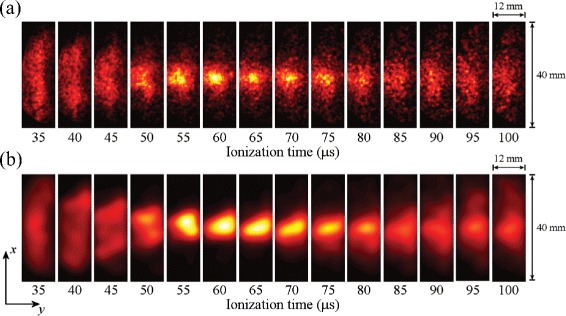
Imaging the transverse motion of H Rydberg atoms in an on-axis electrostatic trap. ‘Breathing’ motion of a trapped ensemble of H Rydberg atoms recorded by imaging the spatial distribution of H ^+^ ions detected at the MCP for selected times after pulsed electric field ionisation. **a** Experimentally recorded data, and (**b**) calculated images. From [118]

In these experiments information on the transverse motion of the ensemble of trapped atoms could be obtained by H ^+^ ion imaging. Such data, together with a corresponding set of calculated ion images are displayed in Fig. 20(a) and (b), respectively. With potentials of ±55 V applied to electrodes 5 and 6, atoms located at the edges of the trap in the *x* dimension when the trap was initially loaded experienced strong electric-field gradients that forced them toward the centre of the trap, compressing the cloud in this dimension. Under the conditions in which the trap was operated this motion of the ensemble had a period of ∼100 *μ*s. This can be seen in the experimental data in Fig. 20(a) which were recorded at time intervals of 5 *μ*s. At early times, the cloud of trapped atoms fills the trap in the *x*-dimension. As the atoms are forced toward the centre of the trap, the spatial spread of the ions impinging on the MCP detector reduces until it reaches its minimal size 60−70 *μ*s after excitation. A similar behaviour is seen in the calculated images, validating the interpretation of the experimental data.

In detecting trapped atoms by pulsed electric field ionisation, the ion time-of-flight distributions can also be exploited to provide information on the location and spatial distribution of the Rydberg atoms at the time of ionisation [113]. By switching off the trap and allowing the cloud of Rydberg atoms to expand before the ionising electric field was applied, the rate of expansion in the *z* dimension could be measured directly. These measurements indicated that the radius of the Rydberg atom cloud increased at a rate of 50 m/s after the trap was switched off. This corresponds to a mean kinetic energy of *Δ* *E*_kin_/*k*_B_∼150 mK of the atoms.

#### Off-axis trap

**Fig. 21:**
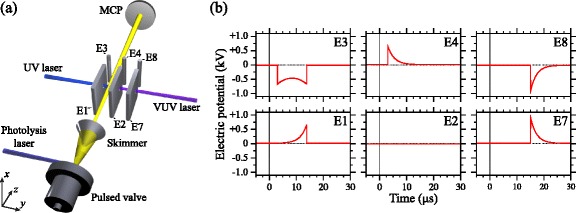
Off-axis electrostatic trap. **a** Schematic diagram of the Rydberg-Stark decelerator and off-axis electrostatic trap (not to scale). In this figure, the end-cap electrodes (E5 and E6, and E9 and E10) which close off the on-axis and off-axis quadrupole traps formed between electrodes E1–E4 and E2, E4, E7 and E8 in the *y* dimension [see Fig. 16(a)] are omitted for clarity. **b** The sequence of electric potentials applied to the six principal electrodes of the device for deceleration and off-axis trapping. The time on the horizontal axes in (**b**) is displayed with respect to the time of photoexcitation. From [120]

To isolate and minimise contributions from collisions of trapped atoms with the trailing components of the atomic beams employed in these experiments, and distinguish collisional losses from trap decay driven by blackbody radiation, an off-axis trap which could be cooled to low-temperatures was developed [105, 120]. The design of this device is presented in Fig. 21(a) as it was initially employed to decelerate beams of H atoms from initial longitudinal speeds of ∼600 m/s. In this decelerator and off-axis trap the laser beams required for Rydberg state photoexcitation entered the excitation region through a set of 1.6-mm-diameter holes in electrodes 1, 2, and 7. Following photoexcitation, a series of pulsed electric potentials were applied to decelerate, deflect and load the Rydberg atoms into the off-axis trap located 6 mm away from the initial propagation axis of the atomic beam.

**Fig. 22:**
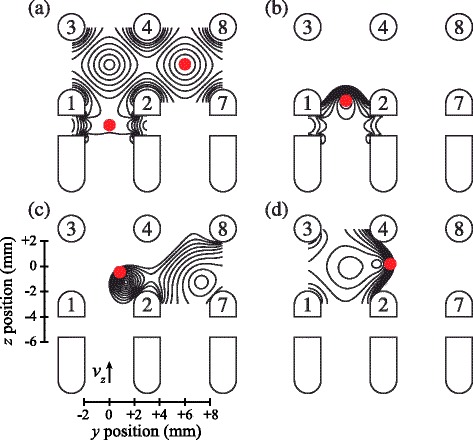
Electric field distributions in the off-axis decelerator and trap. Electric field distributions in the *yz* plane of the off-axis trap (**a**) at the time of photoexcitation and after completion of the trapping process, (**b**) in the initial phase of on-axis deceleration, (**c**) during the 90° deflection process, and (**d**) in the final deceleration phase. The lines of constant electric field range in (**a**) from 10 to 100 V/cm in steps of 10 V/cm, and (**b**–**d**) from 20 to 200 V/cm in steps of 20 V/cm. The red shaded circles indicate the center of the Rydberg atom cloud at each time. From [105]

The sequence of pulsed potentials used for deceleration and trap loading could be tailored to trap atoms on-axis, between electrodes 1–4, or off-axis between electrodes 2, 4, 7, and 8. The pulsed time-dependent potentials required for off-axis trapping can be seen in Fig. 21(b). At the times of photoexcitation and trapping [Fig. 22(b)], potentials of +12 V (-12 V) were applied to electrodes 1, 4, and 7 (2, 3, and 8) forming two electric quadrupole traps in the *yz* plane, with minima located at the midpoint between electrodes 1–4 (on-axis trap) and 2, 4, 7, 8 (off-axis trap). End-cap electrodes [not shown in Fig. 21(a)], were located above (below) the on-axis and off-axis traps, separated by 10 mm in the *x* dimension, and operated at potentials of −22 V (+22 V) to achieve confinement of the Rydberg atom cloud in the *x* direction and generate a field of 10 V/cm at the trap minimum. This represented the electric field configuration for the first of 5 phases of trap loading. After photoexcitation [ *t*=0 *μ*s in Fig. 21(b)], initial longitudinal deceleration took place (0<*t*<5 *μ*s). This was followed by guiding of the atoms off axis (5 *μ*s<*t*<15 *μ*s), transverse deceleration to the position of the off-axis trap (15 *μ*s<*t*<25 *μ*s), and off-axis trapping (*t*≥25 *μ*s). The electric-field distributions in the *yz* plane at the beginning of each of these five phases are displayed in Fig. 22(a-d).

The presence of Rydberg atoms in this off-axis trap was measured by pulsed electric field ionisation following the application of pulsed ionisation potentials of +2 kV to electrodes 2 and 7 with the resulting H ^+^ ions collected on a MCP detector [see Fig. 21(a)]. The results of measurements of this kind for a range of time delays between photoexcitation and pulsed electric field ionisation are presented in Fig. 19 (filled black circles). The data in this figure, all recorded following deceleration and trapping of H atoms initially prepared in low-field-seeing *n*=30 Rydberg-Stark states, permits a direct comparison to be made between the decay of atoms from the on-axis trap with that from the off-axis trap. In these data sets, the decay rates from the two traps are identical at times beyond 170 *μ*s with the difference at early times evidence for collisional losses of ∼40 *%* from the on-axis trap induced by the trailing component of atomic beam. This rapid decay of atoms from the trap at early times is suppressed by trapping off-axis.

Although the rates of decay of electrostatically trapped atoms at later times in Fig. 19 are on the same order of magnitude as those associated with fluorescence from the initially prepared Rydberg-Stark states, no dependence of the decay rate on the value of *n* was observed in this work. This suggested that decay channels other than fluorescence must play a role in the experiments. Because of the sensitivity of the trapped Rydberg atoms to room temperature blackbody radiation, these additional decay channels were attributed to transitions driven by blackbody radiation. However, contributions from Rydberg-Rydberg interactions could also not be excluded at early times.

**Fig. 23:**
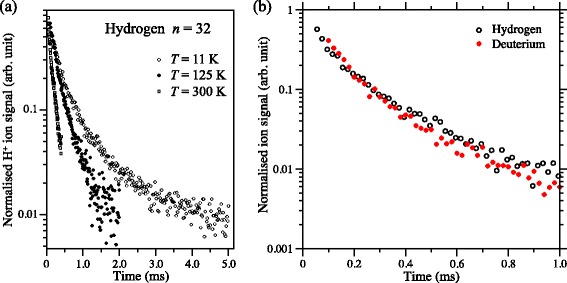
Blackbody temperature, and isotope dependence of trap decay. **a** Temperature dependence of the decay of trapped H atoms initially prepared in *n*=32 Rydberg-Stark states. Experiments were performed at 300 K (*open squares*), 125 K (*black filled circles*), and 11 K (*open circles*). From [151]. **b** Decay of H (*open circles*) and D (*filled circles*) Rydberg atoms, initially prepared in Stark states for which *n*=30, from an off-axis electrostatic trap operated at 125 K. From [120]

The effect of the blackbody temperature of the surroundings of the off-axis trap on the rate of decay of H atoms confined within it can be seen in Fig. 23(a). As the blackbody temperature is reduced from room temperature, to 125 K and 11 K, the period of time for which atoms remain in the trap increases. Consequently, the trap decay rate reduces. Since the strongest transitions induced by blackbody radiation between Rydberg states in the presence of an electric field tend be between states with similar electric dipole moments, such transitions do not lead directly to a loss of atoms from the trap. These transitions lead instead to a gradual redistribution of the Rydberg state population among states with a range of values of *n*. At room temperature, this redistribution renders the measured trapping times largely independent of the value of *n*. Therefore the primary blackbody radiation contribution to the loss of trapped atoms at room temperature in Fig. 23(a) is direct blackbody photoionisation. For states with values of *n* close to 30, photoionisation accounts for approximately 8 *%* of the blackbody-radiation–induced depopulation rate in a 300 K environment [18, 106, 121]. As the blackbody temperature of the environment is reduced the contributions from blackbody photoionisation also reduce. In the data recorded at 125 K and 11 K in Fig. 23(a) the decrease in the decay rate observed at longer trapping times is a result of the slow redistribution of population among states with a range of values of *n* at these temperatures. As the population is redistributed, atoms de-excited to states with lower values of *n* decay more rapidly from the trap. Consequently, at later times only atoms in states with the highest values of *n*, which decay more slowly, remain. At 11 K, the initially prepared states are preserved for ∼500 *μ*s after which time the measured decay rates represent the average rate of decay of the states to which the population has been redistributed following blackbody transitions.

To confirm that the measured rates of decay of atoms from the off-axis trap are dominated by radiative processes and not affected by the dynamics of the trapped atoms, experiments were also performed with D atoms in states with the same values of *n* [120]. A direct comparison between the rate of decay of H and D atoms initially prepared in Rydberg-Stark states for which *n*=30 from an off-axis trap cooled to 125 K can be seen in Fig. 23(b). This indicates that the heavier D atoms can also be efficiently trapped, and the differing dynamics of the ensembles of H and D atoms in the trap, resulting from their slightly different trajectories into the trap, do not significantly affect measurements of their decay.

**Fig. 24:**
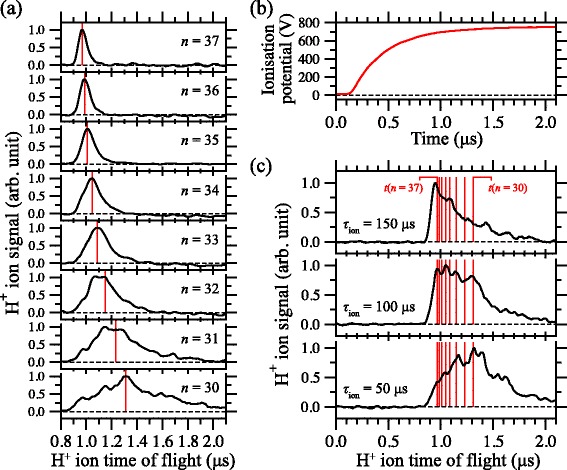
Evolution of Rydberg state populations in a 125 K environment. **a** H ^+^ ion time-of-flight distributions recorded following ramped electric field ionisation 50 *μ*s after trapping H Rydberg atoms initially prepared in Stark states with values of *n* from 30 to 37. **b** The time-dependent ionisation potential applied to electrodes 2 and 7 in Fig. 21(a) to ionise the trapped atoms. **c** Evolution of the ion time-of-flight distribution for atoms initially prepared in states for which *n*=30 as the trapping time is increased in an environment cooled to 125 K. The vertical red bars indicate the ion arrival times for states with consecutive values of *n* as determined from (**a**). From [152]

Evidence for the redistribution of the Rydberg state population toward higher values of *n* at longer trapping times when *T*=125 K can be seen in measurements carried out using slowly-rising ionisation electric-field pulses to project the distribution of Rydberg states onto the H ^+^ ion time-of-flight distribution. The results of such measurements, performed with the trap electrodes and surrounding heat shields cooled to 125 K, are presented in Fig. 24. The set of data in Fig. 24(a) are reference measurements recorded after a trapping time of 50 *μ*s for initially excited Rydberg states with values of *n* ranging from 30 to 37. The slowly-rising electric potential applied to electrodes 2 and 7 to ionise the atoms from the off-axis trap and extract the resulting ions toward the MCP detector is presented in Fig. 24(b). As the ionisation field rises, high-*n* states ionise first (at low fields) while lower *n* states ionise later. The ionisation times are then mapped onto the ion time-of-flight distributions such that ions detected at earlier (later) times correspond to atoms with higher (lower) values of *n*. At 125 K blackbody-radiation–induced transitions do occur during the first 50 *μ*s trapping time. However, although this results in a broadening of the ion time-of-flight distributions, the maximum of each distribution still correspond approximately to the detection time of the initially excited Rydberg state. For increasing values of *n* the maximum of the corresponding ion time-of-flight distribution therefore shifts to earlier times as indicated by the locations of the red vertical lines in Fig. 24(a). From these measurements a H ^+^ ion flight time can be associated with atoms in each *n* state present in the trap. This information can then be employed to investigate the time evolution of a single initially excited Rydberg state as shown for *n*=30 in Fig. 24(c). In this figure, the red vertical lines denote the arrival times of the ions produced by pulsed-field-ionisation of Rydberg states from *n*=30 to *n*=37 as determined from Fig. 24(a). After a trapping time of 50 *μ*s, a significant number of ions arrive at the MCP detector with short flight times indicating the presence of Rydberg states with *n*>30 in the trap. However, the larger fraction of the atoms are still in states for which *n*=30. After a trapping time of 100 *μ*s, an increase in the relative intensity of the signal corresponding to Rydberg states for which *n*>30 is observed. A further increase in this signal is seen after increasing the trapping time to 150 *μ*s. At these longer trapping times, the larger fraction of the trapped atoms are in states with *n*>33. As these higher-*n* states have slower spontaneous emission rates than those of lower *n*, there is a larger probability that they will remain in the trap. This upward shift of the mean value of *n* in the ensemble of trapped atoms with time gives rise to a reduction in the trap-loss rate by spontaneous emission, and the non-exponential decay of atoms from the trap seen in Fig. 19.

### Trapping hydrogen molecules

The phase-space acceptance of the on-axis and off-axis traps described above makes them also well suited to deceleration and trapping molecular hydrogen. By preparing pulsed supersonic beams of H_2_ seeded in Kr, samples with initial kinetic energies equal to those of beams of D atoms can readily be prepared. To decelerate and electrostatically trap these H_2_ molecules hydrogenic Rydberg-Stark states with |*M*_*J*_|=3 were prepared using the three-photon excitation scheme in Eq. (20) [98, 101]. This required the generation of coherent, circularly polarised radiation in the vacuum-ultraviolet (vuv) (94093 cm ^−1^≡106.28 nm), visible (18190 cm ^−1^≡549.75 nm), and infrared (∼1200 cm ^−1^≡833 nm) regions of the electromagnetic spectrum, for each step, respectively. In the experiments in which this photoexcitation scheme has been employed, the visible and infrared laser radiation was directly generated using two tuneable nanosecond pulsed dye lasers. The circularly polarised vuv radiation was generated by resonance enhanced sum-frequency mixing in Xe [98]. By propagating each of these laser beams through 1.5 mm diameter holes in the electrodes surrounding the photoexcitation region the quantisation axis defined by the laser beams coincided with that defined by the electric field at the position of photoexcitation. The use of this excitation geometry ensured the selective photoexcitation of *n*f|*M*_*J*_|=3 Rydberg states converging to the *N* ^+^=0, 2 and 4 rotational states of the $\mathrm {X}\,^{2}\Sigma _{\mathrm {g}}^{+}(v^{+}=0)$ H$_{2}^{+}$ ion core.

The procedure employed to decelerate the H_2_ molecules into the on-axis electrostatic trap is the same as that described above for the deceleration and electrostatic trapping of H Rydberg atoms. However, because the initial longitudinal velocities of the beams of H_2_ generated by seeding in Kr were 500 m/s, and lower than those of the beams of H atoms above, the time constant characterising the exponential decay of the deceleration potentials was increased to 3.65 *μ*s.

**Fig. 25:**
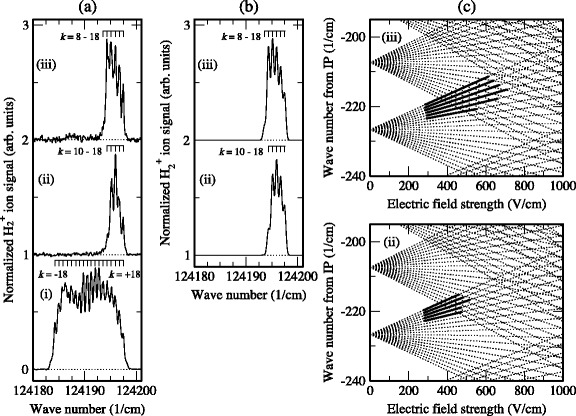
Trapping H_2_ molecules in selected Rydberg-Stark states. (**a**,*i*) *n*=22, |*M*_*J*_|=3 Stark spectrum of H_2_ recorded in an electric field of 278 V/cm with detection after a time delay of 3 *μ*s. (*ii*,*iii*) Spectra of low-field-seeking *k*=10−18, *n*=22, |*M*_*J*_|=3 Stark states of H_2_ detected after a trapping time of 50 *μ*s with deceleration potentials of (*ii*) ±1.7 kV and (*iii*) ±2.3 kV. (**b**,*ii*) and (**b**,*iii*) Calculated spectra obtained following numerical simulations of particle trajectories for deceleration potentials of (*ii*) ±1.7 kV, and (*iii*) ±2.3 kV. **c** Calculated |*M*_*J*_|=3 Stark structure in the vicinity of *n*=22 in H_2_. The thick lines indicate the range of maximum electric-field strength experienced by molecules during deceleration with potentials of (*ii*) ±1.7 kV, and (*iii*) ±2.3 kV. From [98]

In these experiments Rydberg states with a wide range of values of *n* could be decelerated and trapped with any given deceleration pulse sequence. For example, when optimised to decelerate and trap molecules in states for which *n*≃30, states with similar electric dipole moments and values of *n* in the range from *n*=21–37 could also be trapped [101]. For many spectroscopy and scattering experiments, or studies of excited state decay processes, it is desirable to prepare decelerated samples in selected Stark states. As can be seen in Fig. 25, this has been demonstrated in H_2_ for states with *n*=22. Upon photoexcitation in a field of 278 V/cm and detection after a time delay of 3 *μ*s, all 19 accessible *n*=22 Stark states for which |*M*_*J*_|=3, with values of *k* from −18 to +18, are resolved [spectrum (i) of Fig. 25(a)]. Spectra (ii) and (iii) in Fig. 25(a) were then recorded by monitoring the pulsed-field-ionisation signal of the H_2_ molecules in the trap 50 *μ*s after photoexcitation. With pulsed deceleration potentials of ±1.7 kV applied to electrodes 3 and 4 (see Fig. 16), molecules excited to the four outermost low-field-seeking Stark states (*k*=12−18) were trapped efficiently [spectrum (ii)]. Increasing the deceleration potentials to ±2.3 kV enabled the generation of larger electric field gradients and therefore also efficient trapping of molecules in Rydberg-Stark states with *k*=10 [spectrum (iii)], the dipole moment of which was too small for efficient trapping with the lower potentials. The experimentally observed deceleration efficiency for each Stark state is fully accounted for in calculations of particle trajectories in the decelerator and trap, the results of which are displayed in Fig. 25(b). Comparison of the results of these calculations with the experimental data leads to the conclusion that the calculations capture all essential aspects of the deceleration and trapping process, including the dynamics at avoided crossings. Indeed, the range of maximal electric fields experiences by molecules within the excited ensemble during deceleration and trap loading, indicated by the think lines in Fig. 25(c), show that many molecules are subjected to fields beyond the Inglis-Teller limit. The dynamics at the avoided crossings in these fields were treated using a Landau-Zener model in the calculations [98, 122, 123].

**Fig. 26:**
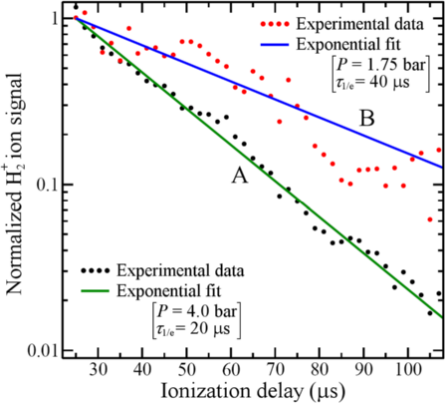
Decay of trapped H_2_ molecules. Measurements of the number of H$^{+}_{2}$ ions detected following pulsed electric field ionisation of H_2_ molecules from an on-axis electrostatic trap in a room temperature environment. Dataset A (B) was recorded with the pulsed valve operated at a stagnation pressure of 4.0 bar (1.75 bar). From [101]

Measurements of the decay of H_2_ molecules from the on-axis electrostatic trap following photoexcitation to |*n*,*k*〉=|33,23〉 Stark states, deceleration and trapping are presented in Fig. 26 [101]. These measurements were performed by monitoring the integrated H$^{+}_{2}$ ion signal at the MCP detector following pulsed electric field ionisation of the trapped molecules for a range of times after photoexcitation. From the data recorded with the lower density molecular beam (dataset B – for which the pulsed valve was operated at a stagnation pressure of 4 bar), a trap decay constant of *τ*_1/e_=40 *μ*s was determined. If spontaneous emission to lower *n* levels, with |*M*_*J*_|=2−4 and hence *n*≥3, were the only decay processes, trapping times exceeding 300 *μ*s would be expected. The significant difference between this time and that measured experimentally indicates that decay by spontaneous emission is not the dominant trap-loss mechanism.

As in the trapping experiments carried out in a room temperature environment with H and D atoms, transitions driven by blackbody radiation can affect the decay of the H_2_ molecules from this on-axis trap. However, while the overall blackbody depopulation rates of the initially prepared Stark states are expected to be similar to those for H or D, the effects of these transitions on the decay of the H_2_ molecules from the trap are different. This is because in the hydrogenic Rydberg-Stark states of H_2_ prepared, blackbody-radiation-induced transitions to |*M*_*J*_|=2 Rydberg states which can decay by predissociation can occur. Under the assumption that approximately one third of the blackbody-radiation-induced transitions are *Δ* *M*_*J*_=−1 transitions one can conclude that ∼33 *%* of the blackbody-radiation-induced depopulation rate results in trap loss, leading to an expected decay time greater than 100 *μ*s.

Because the trap loss rates in Fig. 26 are higher than those expected by the combination of spontaneous emission and blackbody-radiation-induced loss, and a significant dependence of the trap decay rate on the stagnation pressure at which the pulsed valve was operated, and hence the density of the molecular beam, was observed (compare dataset A and dataset B), it must be concluded that collisional losses also play a significant role in these on-axis trapping experiments. In addition, it is also possible that slow predissociation of the |*M*_*J*_|=3 plays a minor part in the trap decay. Collisional losses can occur either through dipole-dipole interactions between trapped Rydberg molecules or via interactions of the Rydberg molecules, with atoms and molecules in the trailing components of the gas pulse.

To disentangle these competing decay processes, experiments have recently been performed by Seiler, Merkt and co-workers with H_2_ molecules trapped in an off-axis electrostatic trap the electrodes of which were cooled to 11 K [124]. This work indicates that, as in the H and D atom trapping experiments, under these conditions H_2_ trapping times exceeding 1 ms are achievable. These experiments now open up a wide range of opportunities for studies of the effects of collisions and blackbody transitions on the decay of long-lived hydrogenic molecular Rydberg states on time-scales that were not previously possible.

## Chip-based guides, decelerators and traps

Controlled manipulation of the motional degrees of freedom and internal quantum states of atoms and molecules at vacuum–solid-state interfaces is of importance in several areas of research. Robust and scaleable chip-based electric and magnetic traps and guides have been developed for atomic ions [125] and neutral ground state atoms [126]. These devices have been exploited, e.g., in quantum information processing [127] and quantum metrology [128]. In addition, the preparation of cold, velocity-controlled samples of polar molecules using chip-based Stark decelerators has been demonstrated as a route “*towards a gas phase molecular laboratory on a chip*” [129–131]. Approaches directed toward the confinement of Rydberg atoms in the vicinity of surfaces have involved Rydberg photoexcitation in miniature vapor cells [49], and in close proximity to arrays of surface-based permanent-magnet traps [132]. Atom chips have also been developed with Rydberg photoexcitation in the strong dipole-blockade regime in mind [133, 134], and for the realisation of sources of single atoms on demand [135].

In this context chip-based Rydberg-Stark decelerators have also recently been developed. These devices are composed of arrays of metallic electrodes on electrically insulating substrates and have been used to accelerate, decelerate and trap pulsed beams of H [136] and He atoms [87, 137], and H_2_ molecules [138] in continuously moving electric traps. Electrostatic trapping at zero mean velocity has also been achieved. These devices are scalable in their construction and therefore well suited to the deceleration of samples with high initial kinetic energies, they can be readily configured to implement complex decelerator or trap geometries, and because the atoms or molecules are always localised about an electric field minimum during deceleration they permit control over the motion of samples in states with a very wide range of principal quantum numbers without losses by electric field ionisation.

**Fig. 27:**
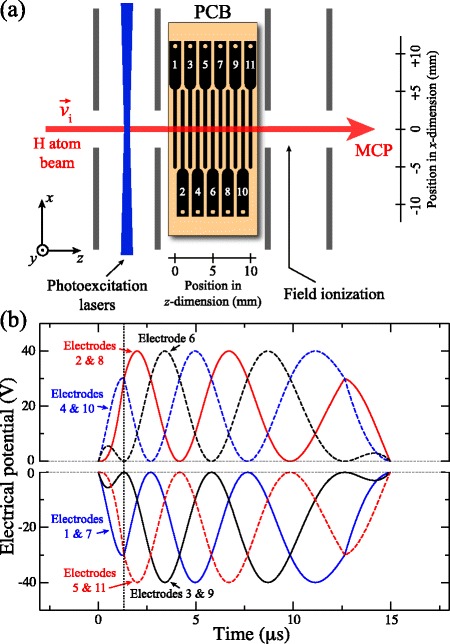
A surface-electrode Rydberg-Stark decelerator. **a** Schematic diagram of a surface-electrode-based Rydberg-Stark decelerator and surrounding photoexcitation and electric field ionisation regions. **b** Oscillating potentials applied to the 11 electrodes of the decelerator in (**a**) for the deceleration of H atoms from *v*_i_=760 m/s to *v*_f_=300 m/s. From [136]

The design and operation principle of the first chip-based decelerator developed for Rydberg atoms and molecules built on those of chip-based Stark decelerators for polar ground state molecules [129, 131]. However, because the electric dipole moments of the Rydberg states employed in the experiments were three orders of magnitude larger than those of the polar molecules decelerated in these other chip-based Stark decelerators (e.g., CO ^∗^ [139]), the electrode dimensions and their spacing could be scaled up, and the amplitudes of the electric potentials applied for deceleration and trapping scaled down, so that entire ensembles of atoms or molecules to be decelerated could be loaded into individual travelling traps of the decelerator. The resulting device, depicted schematically in Fig. 27(a), was composed of 11 electrodes each with a width of 0.5 mm in the *z*-dimension, and a center-to-center spacing *d*_*z*_=1.0 mm. These dimensions were chosen to match the decelerator acceptance to the phase-space characteristics a beam of H Rydberg atoms. As can be seen in Fig. 27(a), the ends of the electrodes in the *x* dimension were enlarged to reduce the oscillatory motion of the minima in this dimension during deceleration.

**Fig. 28:**
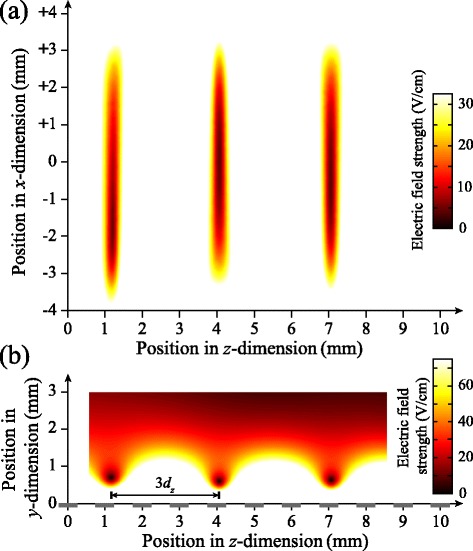
Electric field distributions in a surface-electrode Rydberg-Stark decelerator. Electric-field distribution in (**a**) the *xz*-plane containing the trap minima, and (**b**) the *x*=0 plane in a surface-electrode Rydberg-Stark decelerator. The positions of the decelerator electrodes are indicated on the horizontal axis in (**b**). The 0 V plate is located at *y*=−0.75 mm. From [136]

The decelerator was operated by applying six oscillating electric potentials, *V*_*i*_, to electrodes numbered *i*=1−6 in Fig. 27(a), and repeating the sequence from the seventh electrode on. These potentials have the general form

22 $$\begin{array}{@{}rcl@{}} V_{i}&=&(-1)^{i}\,V_{0}\left[1+\cos(\omega t + \phi_{i})\right], \end{array} $$

where 2*V*_0_ is the peak-to-peak potential, *ω* is the oscillation angular frequency, and *ϕ*_*i*_=(1−*i*)2*π*/3 is the phase shift from one electrode to the next [129]. Using this configuration of time-dependent electric potentials, a set of moving electric traps, separated by a distance of 3*d*_*z*_=3 mm, were generated above the two-dimensional array of electrodes, as depicted in Fig. 28. The activation time of the decelerator potentials was selected to ensure that all of the excited Rydberg atoms were loaded into a single trap. As the potentials oscillated in time, this trap moved in the positive *z* dimension with a velocity *v*_*z*_=3*d*_*z*_ *ω*/(2*π*). Acceleration/deceleration, *a*_*z*_, was achieved by applying a linear frequency chirp to the time-dependent potentials such that

23 $$\begin{array}{@{}rcl@{}} \omega(t)=\omega_{0}+(2\pi/3d_{z})a_{z} t, \end{array} $$

where *ω*_0_ corresponds to the initial velocity *v*_i_. An example of a set of potentials tailored for deceleration from *v*_i_=760 m/s to a final velocity *v*_f_=300 m/s is displayed in Fig. 27(b).

The potentials used to generate the field distributions depicted in Fig. 28 correspond to those at a time of 1.25 *μ*s in Fig. 27(b), at the end of the activation phase (dotted vertical line). For the value of *V*_0_=20 V used here, the trap minimum into which the atoms to be decelerated were loaded was located 0.6 mm above the surface of the device in the *y* dimension. The moving trap was chosen to be ∼5 mm long in the *x* dimension and ∼0.5 mm wide in the *y* and *z* dimensions and the trap depth was ∼27 V/cm, corresponding to *E*/*k*_B_≃2 K (≃5.5 K) for the outer low-field-seeking Stark states at *n*=30 (*n*=50).

**Fig. 29:**
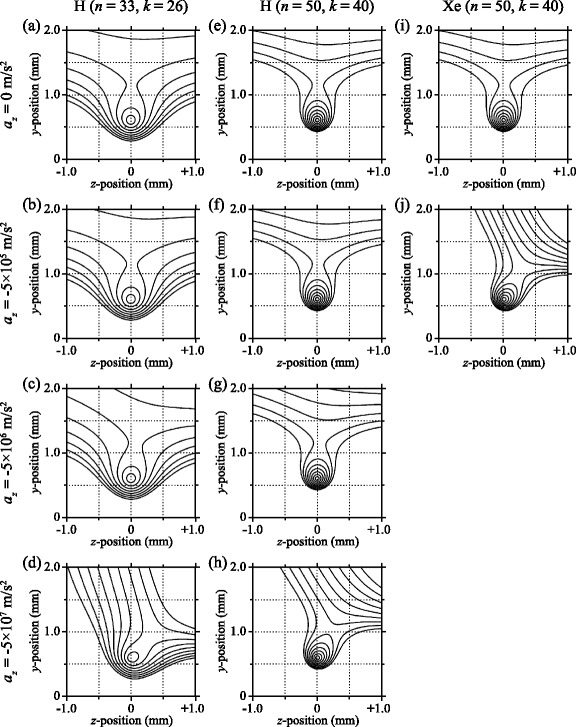
Potential energy distributions in the moving frame of reference associated with an electric field minimum in a surface-electrode Rydberg-Stark decelerator. Potential energy distributions in the *yz*-plane at the mid-point of a surface-electrode Rydberg-Stark decelerator in the *x*-dimension surrounding a moving electric field minimum for accelerations of 0, −5×10^5^, −5×10^6^ and −5×10^7^ m/s^2^. The contour lines are spaced by *E*/*k*_B_=1 K beginning at 1 K. Cases for which H atoms in (**a**–**d**) |*n*,*k*〉=|33,26〉, and (**e**–**h**) |50,40〉 states are displayed. As are those in (**i**) and (**j**) for Xe atoms in the |50,40〉 state

As is the case in the single-stage Rydberg-Stark decelerators described above [120], the acceleration of the continuously moving electric-field minima in these surface-electrode decelerators affects the potential energy distribution in which the trapped Rydberg atoms move. In the surface-electrode decelerator, a constant acceleration is applied as the atoms travel across the device. As a result, the change that this leads to in the potential energy distribution experienced by the atoms in the accelerated frame of reference has a significant effect at all times throughout the acceleration/deceleration process. As in other chip-based Stark decelerators [129, 131] this effect of the acceleration of the moving trap, $\vec {a}_{\text {trap}}$, is most readily seen in the moving frame of reference associated with a single electric field minimum of the decelerator. The transformation from the laboratory-fixed frame of reference to this moving frame is achieved by adding the pseudo potential

24 $$\begin{array}{@{}rcl@{}} V_{\text{pseudo}}=m\,\vec{a}_{\text{trap}}\cdot\vec{s}, \end{array} $$

where *m* is the mass of the atom or molecule being decelerated, and $\vec {s}$ is the displacement from this local field minimum. Potential energy distributions associated with such an electric field minimum in this moving frame of reference of the surface-electrode Rydberg-Stark decelerator are presented in Fig. 29 for accelerations of 0, −5×10^5^, −5×10^6^ and −5×10^7^ m/s^2^. In this figure, the contour lines are spaced by *E*/*k*_B_=1 K and begin at 1 K. For H atoms in |*n*,*k*〉=|33,26〉 states for which the decelerator was designed, the shape and size of the moving trap does not significantly change for accelerations from 0 to −5×10^5^ m/s^2^ [Fig. 29(a) and (b)]. However, the shape of the trap begins to distort for accelerations of −5×10^6^ m/s^2^ and larger [Fig. 29(c)] with a significant reduction in size for an acceleration of −5×10^7^ m/s^2^. For higher Rydberg states with |*n*,*k*〉=|50,40〉, which can also be efficiently decelerated, the moving trap generated using the same electric potentials applied to the electrodes is deeper, and even for an acceleration of −5×10^7^ m/s^2^ is only slightly distorted [Fig. 29(e–h)]. To assess the suitability of such a decelerator for the manipulation of beams of heavy atoms or molecules, the potential energy distributions for Xe atoms in |*n*,*k*〉=|50,40〉 states are presented in Fig. 29(i) and (j) and indicate that accelerations of up to −5×10^5^ m/s^2^ are feasible with this device, making it suitable for the deceleration of heavy samples.

**Fig. 30:**
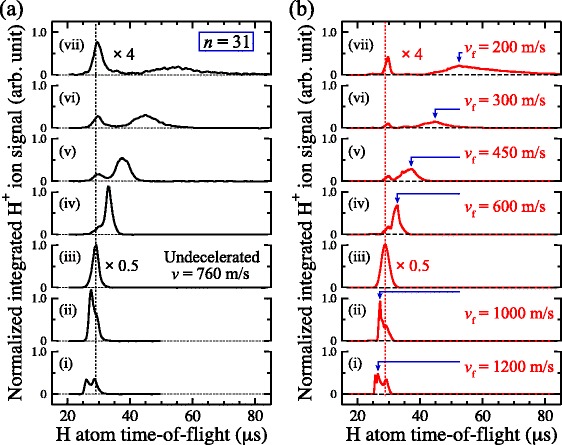
Acceleration and deceleration of H atoms in a surface-electrode Rydberg-Stark decelerator. **a** Experimental, and (**b**) calculated H-atom time-of-flight distributions demonstrating acceleration/deceleration of atoms in states for which *n*=31 from *v*_i_=760 m/s to *v*_f_=1 200, 1 000, 600, 450, 300 and 200 m/s. From [136]

The operation of this surface-electrode decelerator was investigated experimentally by measuring the times-of-flight of beams of H Rydberg atoms from their position of photoexcitation to that of electric field ionisation (see Fig. 27). A set of data corresponding to acceleration/deceleration from *v*_i_=760 m/s to final velocities between 1 200 m/s and 200 m/s, is presented in Fig. 30(a). These measurements only differ in the frequency chirp used to achieve the desired final longitudinal speed. The time-of-flight distribution labelled (iii) corresponds to the undecelerated beam of Rydberg atoms, detected after a flight time of ∼29 *μ*s (vertical dotted line). This measurement was made with the decelerator off and its intensity is scaled by a factor of 0.5 with respect to the other measurements. Measurements (i) and (ii) were performed to accelerate the atoms to *v*_f_=1 200 and 1 000 m/s, respectively. In these cases the accelerated atoms arrived at the detection region earlier than the undecelerated atoms, at times of ∼26 and ∼27.5 *μ*s, respectively. The upper four time-of-flight distributions (iv)–(vii) correspond to deceleration to *v*_f_=600, 450, 300 and 200 m/s and indicate progressively later arrival times of the decelerated atoms of 33, 38, 45 and 54 *μ*s, respectively. The intensity of the distribution corresponding to deceleration to *v*_f_=200 m/s is scaled by a factor of four.

Calculations of three-dimensional particle-trajectories and the corresponding time-of-flight distributions [Fig. 30(b)] capture the essential features of the experimental data and provide a basis for their interpretation. Comparison of the experimental data with the results of the calculations indicate that the peak observed in all traces at ∼29 *μ*s corresponds to atoms which traverse the decelerator at a position beyond the saddle point of the moving traps in the *y* dimension (i.e., at *y*>1.2 mm). These atoms follow metastable trajectories across the decelerator and arrive at the field-ionisation point without acceleration or deceleration. The second peak in each time-of-flight distribution corresponds to the accelerated/decelerated atoms. The velocities at each maximum [indicated by the arrows in Fig. 30(b)] exactly match the final velocities for which the deceleration potentials were designed. At low final velocities, the ensemble of decelerated atoms expand in the longitudinal and transverse dimensions as they fly from the end of the device to the detection region. Transverse expansion after deceleration is accompanied by particle loss and leads to a reduction of the overall signal. This behaviour is also reproduced in the calculations.

**Fig. 31:**
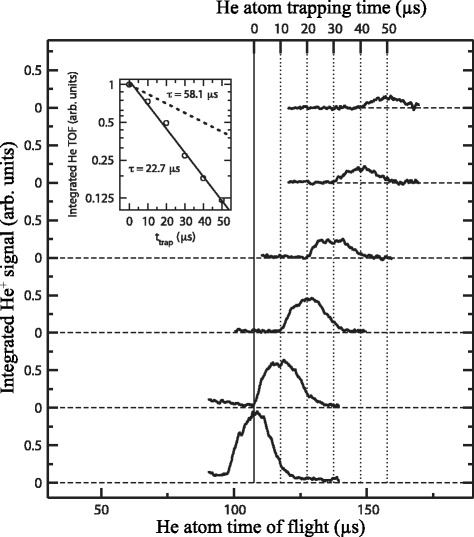
Trapping stationary samples of He atoms in a surface-electrode Rydberg-Stark decelerator. He Rydberg atom time-of-flight distributions after deceleration, trapping, and re-acceleration in a surface-electrode decelerator. The states initially prepared at photoexcitation were those for which |*n*,*k*,*m*〉=|30,23,0〉. The dashed vertical lines indicate the He atom detection times after trapping stationary samples for times ranging from 0 to 50 *μ*s. The dependence of the integrated time-of-flight signal on the trapping time is indicated in the inset, together with the decay rate associated fluorescence alone. From [137] with permission

Following these initial deceleration and trapping experiments this surface-electrode decelerator was extended to include an array of 44 electrodes [137]. This longer device permitted the removal of larger amounts of kinetic energy than in previous decelerators and therefore complete deceleration and trapping of fast beams of He atoms in singlet Rydberg states moving at initial speeds of 1200 m/s. In this work, the deceleration of samples to zero longitudinal velocity in the laboratory-fixed frame of reference, and on-axis trapping was demonstrated in a way that was similar to that employed previously to trap H Rydberg atoms [136]. After the traps in which the atoms were decelerated were brought to a standstill, they remained stationary for a selected period of time, the trapping time, *t*_trap_, in Fig. 31. To detect the trapped atoms by pulsed electric-field ionisation, the traps were subsequently re-accelerated along the axis of the apparatus to 400 m/s and the atoms then released into an electric field ionisation detection region similar to that in Fig. 27. From the complete time-of-flight distributions of the atoms for a set of selected trapping times, information on the decay of atoms from the stationary traps could be determined. The time constants (*τ*_1/*e*_∼25 *μ*s) associated with the observed trap decay in these experiments were shorter than the calculated lifetimes of the states prepared at photoexcitation (*τ*_1/*e*_∼58 *μ*s). This is a result of the combined effects of collisions with the trailing components of the atomic beam arising from on-axis deceleration and trapping, and transitions driven by the room temperature blackbody radiation field in the environment of the decelerator.

**Fig. 32:**
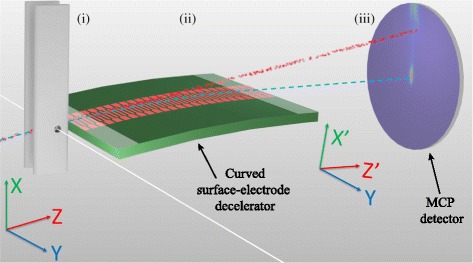
Surface-electrode decelerator and deflector for Rydberg atoms and molecules. Schematic diagram of the surface-electrode decelerator and deflector used to manipulate beams of H_2_ molecules in high Rydberg states. (*i*) The Rydberg state photoexcitation region is located between two parallel metal plates. (*ii*) The curved surface-electrode decelerator permitted controlled transport and deflection of the H_2_ molecules away from their initial axis of propagation. (*iii*) Deflected and undeflected molecules were directly imaged on a MCP detector. From [138] with permission

With a view to using curved, surface-electrode Rydberg-Stark decelerators to prepare velocity-controlled samples of Rydberg atoms and molecules for merged beam collision experiments (see e.g., [140]) devices with curvatures perpendicular to the plane of the electrode surfaces have recently been implemented to transport, decelerate and deflect beams of H_2_ molecules in high Rydberg states [138]. A schematic diagram of the experimental apparatus in which such a device was implemented is displayed in Fig. 32. The H_2_ molecules in these experiments were photoexcited to *n*=31 Rydberg-Stark states between a pair of parallel metallic plates [region (i) in Fig. 32] using the photoexcitation scheme in Eq. 20. The excited molecules were then loaded into a single moving electric trap of the surface-electrode decelerator and transported along the device. In this process the molecules were deflected away from the initial propagation axis of the molecular beam. Images of the undeflected and deflected molecules were then recorded at a MCP detector when the decelerator was off, and when it was activated, respectively. The information obtained from these images was then used to characterise the transverse acceptance of the decelerator and determine the phase-space properties of the guided molecules. An example of such an image, recorded with the decelerator active, is included in Fig. 32. Comparisons of the data recorded in these experiments with the results of numerical calculations of the trajectories of the molecules in the device show excellent agreement. The resulting velocity-controlled beams of cold, state-selected molecules, deflected away from their initial axis of propagation by an angle of 10° had translational temperatures of ∼250 mK and appear very well suited for use in studies of ion-molecule reactions at low collision energies [138].

**Fig. 33:**
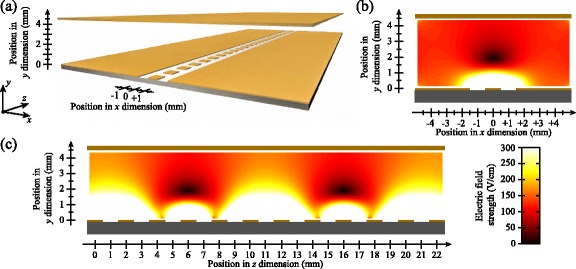
A transmission-line Rydberg-Stark decelerator. **a** Schematic diagram of a transmission-line decelerator for Rydberg atoms and molecules. Typical electric field distributions in (**b**) the transverse *xy*-plane, and (**c**) the longitudinal *yz*-plane employed for trapping, transport and deceleration are also displayed. From [87]

Most recently, a second kind of chip-based Rydberg-Stark decelerator has been developed. This design of this device, which can be seen in Fig. 33, is based on the geometry of a two-dimensional electrical transmission line. This design makes it well suited to integration with chip-based microwave circuits (see e.g., [39]). This transmission-line geometry was first implemented in the form of an electrostatic guide for Rydberg atoms or molecules [141]. This device was composed of an electrical transmission line with a continuous center conductor and permitted the transverse positions of beams of Rydberg atoms to be controlled. By then segmenting the center conductor of the transmission line to form an array of square, equally spaced electrodes, a device in which Rydberg atoms or molecules can be transported, accelerated and decelerated while confined in continuously moving electric traps was realised. The resulting transmission-line decelerator operates on a similar principle to the surface-electrode devices described above, but offers the advantages of stronger transverse confinement, resulting in symmetric traps in all three spatial dimensions [compare Fig. 28 with Fig. 33(b) and (c)], and opportunities for the introduction of curvatures in the plane of the two-dimensional electrode arrays from which it is composed. Because atoms or molecules in this decelerator are conveyed in the void between a two-dimensional electrode array and a parallel plane metal plate it lends itself well to cooling to low temperature, directly shielding the samples within it from their surroundings. It also provides a very well defined electromagnetic environment in which to manipulated and trap Rydberg atoms and molecules. This characteristic is a prerequisite for identifying and precisely controlling effects of blackbody radiation on the trapped samples.

The transmission-line decelerators constructed up to now employ 1 mm square center-conductor segments with center-to-center spacings of *d*_cc_=2 mm. The insulating gap between these segments and the ground planes was also selected to be 1 mm, while the upper plane metal plate was positioned 4.5 mm above this electrode array. In this geometry the formation of a three-dimensional electric field minimum can be achieved by applying equal non-zero electric potentials to one single segment of the center conductor and the upper plate while all other electrodes are set to 0 V. However, if a constant potential of *V*_u_=−*V*_0_/2 is applied to the upper plate electrode, with potentials of 0 V on the two ground planes, and potentials of *V*_*i*_=*V*_0_ cos[−(*i*−1)*ϕ*] on the segments of the center conductor, where *ϕ*=2*π*/5 is the phase-shift from one decelerator segment, *i*, to the next, arrays of electric traps spaced by 5*d*_cc_ are generated within the device.

To realise a continuous motion of this array of traps through the decelerator, the electric potentials applied to the segments of the center conductor are set to oscillate in time at an angular frequency *ω*, such that,

25 $$\begin{array}{@{}rcl@{}} V_{i}(t) = V_{0}\cos\left[\omega t - (i-1)\phi\right]. \end{array} $$

In this configuration the speed, *v*_trap_, at which the traps move through the decelerator is then

26 $$\begin{array}{@{}rcl@{}} v_{\text{trap}} = 5d_{\text{cc}}/(2\pi). \end{array} $$

To ensure that the traps remain a constant distance above the two-dimensional electrode array as they travel through the decelerator the potentials applied to the ground planes are modulated at a frequency, *ω*_gp_=5*ω*, and with an amplitude of −0.045*V*_0_.

**Fig. 34:**
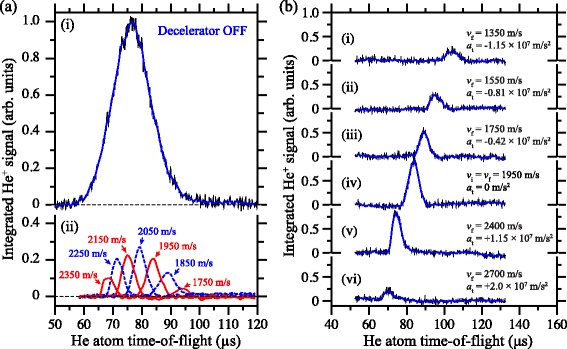
Guiding, accelerating and decelerating He atoms in a transmission-line decelerator. **a** He atom time-of-flight distributions recorded after passing through a transmission-line decelerator (*i*) when the device was off, and (*ii*) after velocity selection and guiding at a range of longitudinal speeds as indicated. **b** Time-of-flight distributions recorded following acceleration/deceleration of atoms travelling with an initial longitudinal speed of 1950 m/s. From [87]

Using a curved decelerator of this design He Rydberg atoms in *n*=52 Stark states with electric dipole moments of ∼6900 D have been velocity selected and guided at constant speed with the results presented in Fig. 34(a). The upper panel, (i), of this figure contains the time-of-flight distribution of the ensemble of initially prepared He Rydberg atoms from their position of photoexcitation to the end of the decelerator with the device off. The results of loading components of this velocity distribution into individual continuously-moving traps of the decelerator, and guiding them through the device at constant speed can be seen in panel (ii). To convey atoms at a constant speed of 1950 m/s through this device required that *ω*=2*π*×195 kHz. In the experiments, these oscillating potentials were generated at low voltage using a set of arbitrary waveform generators before amplification by a factor of 50 to amplitudes of up to *V*_0_=120 V. In the data presented in Fig. 34(a) effects of the Rydberg atom density on the efficiency with which the ensembles are guided can be seen. This highlights the importance of identifying and minimising the effects of collisions between the trapped atoms in experiments of this kind, particularly at these high values of *n* [142].

As in the surface-electrode decelerators discussed above, by introducing frequency chirps to the time-dependent electric potentials, the moving electric traps can be accelerated or decelerated as they travel through the device. For a selected initial speed, *v*_trap_(0), and an acceleration, *a*_trap_, the time-dependence of the oscillation frequency is

27 $$\begin{array}{@{}rcl@{}} \omega(t) = \omega(0) + \frac{\pi a_{\text{trap}}}{5d_{\text{cc}}}t, \end{array} $$

where *ω*(0)=2*π* *v*_trap_(0)/(5*d*_cc_) is the initial angular frequency. Data recorded for the acceleration and deceleration of beams of triplet He atoms in |*n*,*k*〉=|52,35〉 Stark states initially moving at 1950 m/s are presented in Fig. 34(b). As can be seen from this data, increasing the acceleration of the traps toward |*a*_trap_|=2×10^7^ m/s^2^ leads to a reduction in the intensity of the time-of-flight signals. This is a consequence of the effective reduction in the depths of the moving traps with acceleration as discussed above in the context of the surface-electrode decelerators (see, e.g., Fig. 29). In the curved transmission-line decelerator employed in these experiments it was necessary to consider the combined effects of the applied tangential acceleration, and the centripetal accelerations arising from the curvature of the device, on the decelerating atoms. For the Rydberg states prepared in the experiments, the maximum acceleration that could be applied before the traps opened completely was |*a*_trap_|≃2.35×10^7^ m/s^2^.

In these transmission-line decelerators, atomic beams with speeds exceeding 2500 m/s have been manipulated and changes in kinetic energy of up to *E*_kin_∼1.3×10^−20^ J (≡80 meV or ≡650 cm ^−1^) have been achieved. This makes them very well suited to the transport, acceleration and trapping of a wide range of atoms and molecules. At present transmission-line decelerators with *in situ* detection are being developed and tested, and possibilities for the implementation of chip-based storage-rings, and beam-splitters are foreseen. These devices, when combined with co-planar microwave circuitry represent a unique chip-based laboratory with which comprehensive control over the translational motion and internal quantum states of gas-phase Rydberg atoms and molecules can be achieved.

## Conclusions

The methods for accelerating, decelerating and trapping Rydberg atoms and molecules initially travelling in pulsed supersonic beams using inhomogeneous electric fields that are reviewed here, open many exciting possibilities for new experimental investigations at the interface between physics and physical chemistry. In the process of developing these techniques measurements have been made of the roles that collisions and transitions driven by blackbody radiation play on the evolution and decay of excited states of atoms and molecules on timescales that were not previously possible. The translationally cold, velocity-controlled beams that can now be prepared by Rydberg-Stark deceleration offer opportunities for investigations of effects of collisions, blackbody radiation and electric and magnetic fields on slow-dissociation processes in long-lived Rydberg states of molecules, and provide an intriguing route to studies of ion-molecule reactions in merged beams [138]. Tuneable, velocity-controlled beams of atoms and molecules in high Rydberg states provide opportunities for studies of the effects of image states, and the velocity dependence of surface-ionisation processes [56]. In addition, the de-excitation of molecular samples back to their ground states after Rydberg-Stark deceleration represents a route toward to the production of cold samples of ground-state molecules.

In hybrid approaches to quantum information processing involving Rydberg atoms and microwave circuits, the chip-based Rydberg atom guides and decelerators described can be used to transport atoms within cryogenic environments operated a different blackbody temperatures, and with differing cooling capacities. Recent implementations of electrostatic charged-wire guides for atoms or molecules in high-field-seeking Rydberg-Stark states [143] offer potential opportunities for studies of angular-momentum quantisation in non-commutative space [144–146]. The demonstrations of focussing, decelerating and trapping fast-moving beams of Rydberg atoms highlight the possibility of employing Rydberg-Stark deceleration methods in the manipulation of samples of positronium atoms for precision spectroscopic studies and investigations of the acceleration of particles composed of antimatter in the gravitational field of the Earth [80].

In the refinement of the techniques of Rydberg-Stark deceleration and electric trapping for application in each of the above areas, it will be important to study and account for the effects of electric-field noise on the energy level structure of the Rydberg states with their very large electric dipole moments [142, 147]. In addition, contributions from dipole-dipole interactions [58, 142, 148, 149] between atoms and molecules during deceleration and trapping must be expected to become increasingly significant particularly on longer experimental timescales.

## References

[1] Rydberg JR (1890). Über den Bau der Linienspektren der chemischen Grundstoffe. Z Phys Chem.

[2] Merkt F (1997). Molecules in high Rydberg states. Ann Rev Phys Chem.

[3] Softley TP (2004). Applications of molecular Rydberg states in chemical dynamics and spectroscopy. Int Rev Phys Chem.

[4] Pauli W (1926). Über das Wasserstoffspektrum vom Standpunkt der neuen Quantunmechanik. Z Phys.

[5] Damburg RJ, Kolosov VV, Stebbings RF, Dunning FB (1983). Theoretical studies of hydrogen Rydberg atoms in electric fields. Rydberg States of Atoms and Molecules.

[6] Osterwalder A, Merkt F (1999). Using high Rydberg states as electric field sensors. Phys Rev Lett.

[7] Bennewitz HG, Paul W, Schlier Ch (1955). Fokussierung polarer Moleküle. Z Phys.

[8] Gordon JP, Zeiger HJ, Townes CH (1955). The Maser – New type of microwave amplifier, frequency standard, and spectrometer. Phys Rev.

[9] Brooks PR (1976). Reactions of oriented molecules. Science.

[10] Stolte S (1982). Reactive scattering studies on oriented molecules. Ber Bunsenges Phys Chem.

[11] Parker DH, Bernstein RB (1989). Oriented molecule beams via the electrostatic hexapole: Preparation, characterization, and reactive scattering. Ann Rev Phys Chem.

[12] Bethlem HL, Berden G, Meijer G (1999). Decelerating neutral dipolar molecules. Phys Rev Lett.

[13] Bethlem HL, Berden G, Crompvoets FMH, Jongma RT, van Roij AJA, Meijer G (2000). Electrostatic trapping of ammonia molecules. Nature.

[14] van de Meerakker SYT, Bethlem HL, Vanhaecke N, Meijer G (2012). Manipulation and control of molecular beams. Chem Rev.

[15] Gerlach W, Stern O (1921). Der experimentelle Nachweis des magnetischen Moments des Silberatoms. Z Phys.

[16] Gerlach W, Stern O (1922). Der experimentelle Nachweis der Richtungsquantelung im Magnetfeld. Z Phys.

[17] Gerlach W, Stern O (1922). Das magnetische Moment des Silberatoms. Z Phys.

[18] Gallagher TF (1994). Rydberg Atoms.

[19] Shuman ES, Barry JF, DeMille D (2010). Laser cooling of a diatomic molecule. Nature.

[20] Zhelyazkova V, Cournol A, Wall TE, Matsushima A, Hudson JJ, Hinds EA, Tarbutt MR, Sauer BE (2014). Laser cooling and slowing of CaF molecules. Phys Rev A.

[21] Vanhaecke N, Meier U, Andrist M, Meier BH, Merkt F (2007). Multistage Zeeman deceleration of hydrogen atoms. Phys Rev A.

[22] Hogan SD, Sprecher D, Andrist M, Vanhaecke N, Merkt F (2007). Zeeman deceleration of H and D. Phys Rev A.

[23] Narevicius E, Libson A, Parthey CG, Chavez I, Narevicius J, Even U, Raizen MG (2008). Stopping supersonic beams with a series of pulsed electromagnetic coils: An atomic coilgun. Phys Rev Lett.

[24] Al-Ramadhan AH, Gidley DW (1994). New precision measurement of the decay rate of singlet positronium. Phys Rev Lett.

[25] Vallery RS, Zitzewitz PW, Gidley DW (2003). Resolution of the orthopositronium-lifetime puzzle. Phys Rev Lett.

[26] Bell MT, Softley TP (2009). Ultracold molecules and ultracold chemistry. Mol Phys.

[27] Carr LC, DeMille D, Krems RV, Ye J (2009). Cold and ultracold molecules: Science, technology and applications. New J Phys.

[28] Fulton R, Bishop AI, Barker PF (2004). Optical Stark decelerator for molecules. Phys Rev Lett.

[29] Messer JK, De Lucia FC (1984). Measurement of pressure-broadening parameters for the CO-He system at 4 K. Phys Rev Lett.

[30] Doyle JM, Friedrich B, Kim J, Patterson D (1995). Buffer-gas loading of atoms and molecules into a magnetic trap. Phys Rev A.

[31] Willitsch S, Bell MT, Gingell AD, Procter SR, Softley TP (2008). Cold reactive collisions between laser-cooled ions and velocity-selected neutral molecules. Phys Rev Lett.

[32] Staanum PF, Højbjerre K, Wester R, Drewsen M (2008). Probing isotope effects in chemical reactions using single ions. Phys Rev Lett.

[33] Choi JH, Guest JR, Povilus AP, Hansis E, Raithel G (2005). Magnetic trapping of long-lived cold Rydberg atoms. Phys Rev Lett.

[34] Amoretti M, Amsler C, Bonomi G, Bouchta A, Bowe P, Carraro C, Cesar CL, Charlton M, Collier MJT, Doser M, Filippini V, Fine KS, Fontana A, Fujiwara MC, Funakoshi R, Genova P, Hangst JS, Hayano RS, Holzscheiter MH, Jorgensen LV, Lagomarsino V, Landua R, Lindelof D, Rizzini EL, Macri M, Madsen N, Manuzio G, Marchesotti M, Montagna P, Pruys H, Regenfus C, Riedler P, Rochet J, Rotondi A, Rouleau G, Testera G, Variola A, Watson TL, van der Werf DP (2002). Production and detection of cold antihydrogen atoms. Nature.

[35] Anderson SE, Younge KC, Raithel G (2011). Trapping Rydberg atoms in an optical lattice. Phys Rev Lett.

[36] Stepkin SV, Konovalenko AA, Kantharia NG, Udaya Shankar N (2007). Radio recombination lines from the largest bound atoms in space. Mon Not R Astron Soc.

[37] Wayne RP (1993). Photodissociation dynamics and atmospheric chemistry. J Geophys Res.

[38] Merkt F, Osterwalder A (2002). Millimeter wave spectroscopy of high Rydberg states. Int Rev Phys Chem.

[39] Hogan SD, Agner JA, Merkt F, Thiele T, Filipp S, Wallraff A (2012). Driving Rydberg-Rydberg transitions from a coplanar microwave waveguide. Phys Rev Lett.

[40] Thiele T, Filipp S, Agner JA, Schmutz H, Deiglmayr J, Stammeier M, Allmendinger P, Merkt F, Wallraff A (2014). Manipulating Rydberg atoms close to surfaces at cryogenic temperatures. Phys Rev A.

[41] Osterwalder A, Wüest A, Merkt F, Jungen Ch (2004). High-resolution millimeter wave spectroscopy and multichannel quantum defect theory of the hyperfine structure in high Rydberg states of molecular hydrogen H_2_. J Chem Phys.

[42] Liu J, Salumbides EJ, Hollenstein U, Koelemeij JCJ, Eikema KSE, Ubachs W, Merkt F (2009). Determination of the ionization and dissociation energies of the hydrogen molecule. J Chem Phys.

[43] Liu J, Sprecher D, Jungen Ch, Ubachs W, Merkt F (2010). Determination of the ionization and dissociation energies of the deuterium molecule (D_2_). J Chem Phys.

[44] Sprecher D, Liu J, Jungen Ch, Ubachs W, Merkt F (2010). The ionization and dissociation energies of HD. J Chem Phys.

[45] Ramsey NF (1950). A molecular beam resonance method with separated oscillating fields. Phys Rev.

[46] Henson AB, Gersten S, Shagam Y, Narevicius J, Narevicius E (2012). Observation of resonances in Penning ionization reactions at sub-Kelvin temperatures in merged beams. Science.

[47] Shagam Y, E. Narevicius E (2013). Sub-Kelvin collision temperatures in merged neutral beams by correlation in phase-space. J Phys Chem C.

[48] Hinds EA, Lai KS, Schnell M (1997). Atoms in micron-sized metallic and dielectric waveguides. Phil Trans R Soc Lond A.

[49] Kübler H, Shaffer JP, Baluktsian T, Löw R, Pfau T (2010). Coherent excitation of Rydberg atoms in micrometre-sized atomic vapour cells. Nat Photon.

[50] Gray DF, Zheng Z, Smith KA, Dunning FB (1988). Ionization of K(*n*d) Rydberg-state atoms at a surface. Phys Rev A.

[51] Anderson A, Haroche S, Hinds EA, Jhe W, Meschede D (1988). Measuring the van der Waals forces between a Rydberg atom and a metallic surface. Phys Rev A.

[52] Sandoghdar V, Sukenik CI, Hinds EA, Haroche S (1992). Direct measurement of the van der Waals interaction between an atom and its images in a micron-sized cavity. Phys Rev Lett.

[53] Hill SB, Haich CB, Zhou Z, Nordlander P, Dunning FB (2000). Ionization of xenon Rydberg atoms at a metal surface. Phys Rev Lett.

[54] Lloyd GR, Procter SR, Softley TP (2005). Ionization of hydrogen Rydberg molecules at a metal surface. Phys Rev Lett.

[55] So E, Dethlefsen M, Ford M, Softley TP (2011). Charge transfer of Rydberg H atoms at a metal surface. Phys Rev Lett.

[56] Gibbard JA, Dethlefsen M, Kohlhoff M, Rennick CJ, So E, Ford M, Softley TP (2015). Resonant charge transfer of hydrogen Rydberg atoms incident on a Cu(100) projected band-gap surface. Phys Rev Lett.

[57] Gallagher TF, Pillet P (2008). Dipole-dipole interactions of Rydberg atoms. Adv At Mol Opt Phys.

[58] Comparat DP, Pillet P (2010). Dipole blockade in a cold Rydberg atomic sample. J Opt Soc Am B.

[59] Anderson WR, Veale JR, Gallagher TF (1998). Resonant dipole-dipole energy transfer in a nearly frozen Rydberg gas. Phys Rev Lett.

[60] Mourachko I, Comparat D, de Tomasi F, Fioretti A, Nosbaum P, Akulin VM, Pillet P (1998). Many-body effects in a frozen Rydberg gas. Phys Rev Lett.

[61] Vogt T, Viteau M, Zhao J, Chotia A, Comparat D, Pillet P (2006). Dipole blockade at Förster resonances in high resolution laser excitation of Rydberg states of cesium atoms. Phys Rev Lett.

[62] Pritchard JD, Maxwell D, Gauguet A, Weatherill KJ, Jones MPA, Adams CS (2010). Cooperative atom-light interaction in a blockaded Rydberg ensemble. Phys Rev Lett.

[63] Wilk T, Gaëtan A, Evellin C, Wolters J, Miroshnychenko Y, Grangier P, Browaeys A (2010). Entanglement of two individual neutral atoms using Rydberg blockade. Phys Rev Lett.

[64] Isenhower L, Urban E, Zhang XL, Gill AT, Henage T, Johnson TA, Walker TG, Saffman M (2010). Demonstration of a neutral atom controlled-NOT quantum gate. Phys Rev Lett.

[65] Smith KA, Kellert FG, Rundel RD, Dunning FB, Stebbings RF (1978). Discrete energy transfer in collisions of Xe(*n*f) Rydberg atoms with NH_3_ molecules. Phys Rev Lett.

[66] Greene CH, Dickinson AS, Sadeghpour HR (2000). Creation of polar and nonpolar ultra-long-range Rydberg molecules. Phys Rev Lett.

[67] Bendkowsky V, Butscher B, Nipper J, Shaffer JP, Löw R, Pfau T (2009). Observation of ultralong-range Rydberg molecules. Nature.

[68] Walther H, Varcoe BTH, Englert BG, Becker T (2006). Cavity quantum electrodynamics. Rep Prog Phys.

[69] Wallraff A, Schuster DI, Blais A, Frunzio L, Huang RS, Majer J, Kumar S, Girvin SM, Schoelkopf RJ (2004). Strong coupling of a single photon to a superconducting qubit using circuit quantum electrodynamics. Nature.

[70] Rabl P, DeMille D, Doyle JM, Lukin MD, Schoelkopf RJ, Zoller P (2006). Hybrid quantum processors: Molecular ensembles as quantum memory for solid state circuits. Phys Rev Lett.

[71] Carter JD, Cherry O, Martin JDD (2012). Electric-field sensing near the surface microstructure of an atom chip using cold Rydberg atoms. Phys Rev A.

[72] Hermann-Avigliano C, Teixeira RC, Nguyen TL, Cantat-Moltrecht T, Nogues G, Dotsenko I, Gleyzes S, Raimond JM, Haroche S, Brune M (2014). Long coherence times for Rydberg qubits on a superconducting atom chip. Phys Rev A.

[73] Humberston JW, Charlton M, Jacobson FM, Deutch BI (1987). On antihydrogen formation in collisions of antiprotons with positronium. J Phys B: At Mol Opt Phys.

[74] Charlton M (1990). Antihydrogen production in collisions of antiprotons with excited states of positronium. Phys Lett A.

[75] Storry CH, Speck A, Sage DL, Guise N, Gabrielse G, Grzonka D, Oelert W, Schepers G, Sefzick T, Pittner H, Herrmann M, Walz J, Hänsch TW, Comeau D, Hessels EA (2004). First laser-controlled antihydrogen production. Phys Rev Lett.

[76] Kellerbauer A, Amoretti M, Belov AS, Bonomi G, Boscolo I, Brusa RS, Büchner M, Byakov VM, Cabaret L, Canali C, Carraro C, Castelli F, Cialdi S, de Combarieu M, Comparat D, Consolati G, Djourelov N, Doser M, Drobychev G, Dupasquier A, Ferrari G, Forget P, Formaro L, Gervasini A, Giammarchi MG, Gninenko SN, Gribakin G, Hogan SD, Jacquey M, Lagomarsino V, Manuzio G, Mariazzi S, Matveev VA, Meier JO, Merkt F, Nedelec P, Oberthaler MK, Pari P, Prevedelli M, Quasso F, Rotondi A, Sillou D, Stepanov SV, Stroke HH, Testera G, Tino GM, Trénec G, Vairo A, Vigué J, Walters H, Warring U, Zavatarelli S, Zvezhinskij DS (2008). Proposed antimatter gravity measurement with an antihydrogen beam. Nucl Instr Meth Phys Res B.

[77] Andresen GB, Ashkezari MD, Baquero-Ruiz M, Bertsche W, Bowe PD, Butler E, Cesar CL, Chapman S, Charlton M, Deller A, Eriksson S, Fajans J, Friesen T, Fujiwara MC, Gill DR, Gutierrez A, Hangst JS, Hardy WN, Hayden ME, Humphries AJ, Hydomako R, Jenkins MJ, Jonsell S, Jørgensen LV, Kurchaninov L, Madsen N, Menary S, Nolan P, Olchanski K, Olin A, Povilus A, Pusa P, Robicheaux F, Sarid E, Seif el Nasr S, Silveira DM, So C, Storey JW, Thompson RI, van der Werf DP, Wurtele JS, Yamazaki Y (2010). Trapped antihydrogen. Nature.

[78] Andresen GB, Ashkezari MD, Baquero-Ruiz M, Bertsche W, Bowe PD, Butler E, Cesar CL, Charlton M, Deller A, Eriksson S, Fajans J, Friesen T, Fujiwara MC, Gill DR, Gutierrez A, Hangst JS, Hardy WN, Hayano RS, Hayden ME, Humphries AJ, Hydomako R, Jonsell S, Kemp SL, Kurchaninov L, Madsen N, Menary S, Nolan P, Olchanski K, Olin A, Pusa P, Rasmussen CØ, Robicheaux F, Sarid E, Silveira DM, So C, Storey JW, Thompson RI, van der Werf DP, Wurtele JS, Yamazaki Y (2011). Confinement of antihydrogen for 1000 seconds. Nat Phys.

[79] Mills Jr AP, Leventhal M (2002). Can we measure the gravitational free fall of cold Rydberg state positronium?. Nucl Inst Meth Phys Res B.

[80] Cassidy DB, Hogan SD (2014). Atom control and gravity measurements using Rydberg positronium. Int J Mod Phys Conf Ser.

[81] Karshenboim SG (2005). Precision physics of simple atoms: QED tests, nuclear structure and fundamental constants. Phys Rep.

[82] Wall TE, Alonso AM, Cooper BS, Deller A, Hogan SD, Cassidy DB (2015). Selective production of Rydberg-Stark states of positronium. Phys Rev Lett.

[83] Ziock KP, Howell RH, Magnotta F, Failor RA, Jones KM (1990). First observation of resonant excitation of high-*n* states in positronium. Phys Rev Lett.

[84] Cassidy DB, Hisakado TH, Tom HWK, Mills AP (2012). Efficient production of Rydberg positronium. Phys Rev Lett.

[85] Hogan SD (2013). Calculated photoexcitation spectra of positronium Rydberg states. Phys Rev A.

[86] Crivelli P, Gendotti U, Rubbia A, Liszkay L, Perez P, Corbel C (2010). Measurement of the orthopositronium confinement energy in mesoporous thin films. Phys Rev A.

[87] Lancuba P, Hogan SD (2014). Transmission-line decelerators for atoms in high Rydberg states. Phys Rev A.

[88] Bethe HA, Salpeter EE (1957). Quantum mechanics of one- and two-electron atoms.

[89] Englefield MJ (1972). Group theory and the Coulomb problem.

[90] Kleppner D, Littman MG, Zimmerman ML, Stebbings RF, Dunning FB (1983). Rydberg atoms in strong fields. Rydberg States of Atoms and Molecules.

[91] Foot C (2005). Atomic physics.

[92] Hiskes JR, Tarter CB, Moody DA (1964). Stark lifetimes for the hydrogen atom. Phys Rev A.

[93] Zimmerman ML, Littman MG, Kash MM, Kleppner D (1979). Stark structure of the Rydberg states of alkali-metal atoms. Phys Rev A.

[94] Fielding HH, Softley TP (1991). Observation of the Stark effect in autoionising Rydberg states of molecular hydrogen. Chem Phys Lett.

[95] Qin K, Bistransin M, Glab WL (1993). Stark effect and rotational-series interactions on high Rydberg states of molecular hydrogen. Phys Rev A.

[96] Vrakking MJ (1996). Lifetimes of Rydberg states in ZEKE experiments. III. Calculations of the dc electric field dependence of predissociation lifetimes of NO. J Chem Phys.

[97] Zare RN (1988). Angular Momentum.

[98] Seiler Ch, Hogan SD, Merkt F (2011). Trapping cold molecular hydrogen. Phys Chem Chem Phys.

[99] Yamakita Y, Procter SR, Goodgame AL, Softley TP, Merkt F (2004). Deflection and deceleration of hydrogen Rydberg molecules in inhomogeneous electric fields. J Chem Phys.

[100] Wall TE, Cassidy DB, Hogan SD (2014). Single-color two-photon spectroscopy of Rydberg states in electric fields. Phys Rev A.

[101] Hogan SD, Seiler Ch, Merkt F (2009). Rydberg-state-enabled deceleration and trapping of cold molecules. Phys Rev Lett.

[102] Hollberg L, Hall JL (1984). Measurement of the shift of Rydberg energy levels induced by blackbody radiation. Phys Rev Lett.

[103] Beiting EJ, Hildebrandt GF, Kellert FG, Foltz GW, Smith KA, Dunning FB, Stebbings RF (1979). The effects of 300 K background radiation on Rydberg atoms. J Chem Phys.

[104] Gallagher TF, Cooke WE (1979). Interactions of blackbody radiation with atoms. Phys Rev Lett.

[105] Seiler Ch, Hogan SD, Schmutz H, Agner JA, Merkt F (2011). Collisional and radiative processes in adiabatic deceleration, deflection and off-axis trapping of a Rydberg atom beam. Phys Rev Lett.

[106] Spencer WP, Vaidyanathan AG, Kleppner D, Ducas TW (1982). Photoionization by blackbody radiation. Phys Rev A.

[107] Mandel M (1979). Photon occupation numbers in black body radiation. J Opt Soc Am.

[108] Wing WH (1980). Electrostatic trapping of neutral atomic particles. Phys Rev Lett.

[109] Breeden T, Metcalf H (1981). Stark acceleration of Rydberg atoms in inhomogeneous electric fields. Phys Rev Lett.

[110] Townsend D, Goodgame AL, Procter SR, Mackenzie SR, Softley TP (2001). Deflection of krypton Rydberg atoms in the field of an electric dipole. J Phys B At Mol Opt Phys.

[111] Procter SR, Yamakita Y, Merkt F, Softley TP (2003). Controlling the motion of hydrogen molecules. Chem Phys Lett.

[112] Vliegen E, Wörner HJ, Softley TP, Merkt F (2004). Nonhydrogenic effects in the deceleration of Rydberg atoms in inhomogeneous electric fields. Phys Rev Lett.

[113] Vliegen E, Merkt F (2006). Stark deceleration of hydrogen atoms. J Phys B: At Mol Opt Phys.

[114] Vliegen E, Merkt F (2005). On the electrostatic deceleration of argon atoms in high Rydberg states by time-dependent inhomogeneous electric fields. J Phys B At Mol Opt Phys.

[115] Vliegen E, Limacher P, Merkt F (2006). Measurement of the three-dimensional velocity distribution of Stark-decelerated Rydberg atoms. Eur Phys J D.

[116] Vliegen E, Merkt F (2006). Normal-incidence electrostatic Rydberg atom mirror. Phys Rev Lett.

[117] Vliegen E, Hogan SD, Schmutz H, Merkt F (2007). Stark deceleration and trapping of hydrogen Rydberg atoms. Phys Rev A.

[118] Hogan SD, Merkt F (2008). Demonstration of three-dimensional electrostatic trapping of state-selected Rydberg atoms. Phys Rev Lett.

[119] Willitsch S, Dyke JM, Merkt F (2003). Generation and high-resolution photoelectron spectroscopy of small organic radicals in cold supersonic expansions. Helv Chim Acta.

[120] Hogan SD, Seiler Ch, Merkt F (2013). Motional, isotope and quadratic Stark effects in Rydberg-Stark deceleration and off-axis electric trapping of H and D. J Phys B At Mol Opt Phys.

[121] Beterov II, Tretyakov DB, Ryabtsev II, Entin VM, Ekers A, Bezuglov NN (2009). Ionization of Rydberg atoms by blackbody radiation. New J Phys.

[122] Landau LD (1932). Zur Theorie der Energieübertragung ii. Phys Z Sowjetunion.

[123] Zener C (1932). Non-adiabatic crossing of energy levels. Proc R Soc London Ser A.

[124] Seiler Ch (2013). Rydberg-Stark deceleration and trapping of atoms and molecules. PhD thesis.

[125] Chiaverini J, Blakestad RB, Britton J, Jost JD, Langer C, Leibfried D, Ozeri R, Wineland DJ (2005). Surface-electrode architecture for ion-trap quantum information processing. Quant Inf Comput.

[126] Folman R, Krüger P, Schmiedmayer J, Denschlag J, Henkel C (2002). Microscopic atom optics: From wires to an atom chip. Adv At Mol Opt Phys.

[127] Home JP, Hanneke D, Jost JD, Amini JM, Leibfried D, Wineland DJ (2009). Complete methods set for scalable ion trap quantum information processing. Science.

[128] Riedel MF, Böhi P, Li Y, Hänsch TW, Sinatra A, Treutlein P (2010). Atom-chip-based generation of entanglement for quantum metrology. Nature.

[129] Meek SA, Bethlem HL, Conrad H, Meijer G (2008). Trapping molecules on a chip in traveling potential wells. Phys Rev Lett.

[130] Meek SA, Santambrogio G, Conrad H, Meijer G (2009). Taming molecular beams; towards a gas-phase molecular laboratory on a chip. J Phys Conf Ser.

[131] Meek SA, Conrad H, Meijer G (2009). Trapping molecules on a chips. Science.

[132] Tauschinsky A, Thijssen RMT, Whitlock S, van Linden van den Heuvell HB, Spreeuw RJC (2010). Spatially resolved excitation of Rydberg atoms and surface effects on an atom chip. Phys Rev A.

[133] Nirrengarten T, Qarry A, Roux C, Emmert A, Nogues G, Brune M, Raimond JM, Haroche S (2006). Realization of a superconducting atom chip. Phys Rev Lett.

[134] Cherry O, Carter JD, Martin JDD (2009). An atom chip for the manipulation of ultracold atoms. Can J Phys.

[135] Saffman M, Walker TG (2002). Creating single-atom and single-photon sources from entangled atomic ensembles. Phys Rev A.

[136] Hogan SD, Allmendinger P, Saßmannshausen H, Schmutz H, Merkt F (2012). Surface-electrode Rydberg-Stark decelerator. Phys Rev Lett.

[137] Allmendinger P, Agner JA, Schmutz H, Merkt F (2013). Deceleration and trapping of a fast supersonic beam of metastable helium atoms with a 44-electrode chip decelerator. Phys Rev A.

[138] Allmendinger P, Deiglmayr J, Agner JA, Schmutz H, Merkt F (2014). Surface-electrode decelerator and deflector for Rydberg atoms and molecules. Phys Rev A.

[139] Santambrogio G (2015). Trapping molecules on chips. EPJ Tech. Instrum..

[140] Osterwalder A (2015). Merged neutral beams. EPJ Tech Instrum.

[141] Lancuba P, Hogan SD (2013). Guiding Rydberg atoms above surface-based transmission lines. Phys Rev A.

[142] Zhelyazkova V, Hogan SD (2015). Probing interactions between Rydberg atoms with large electric dipole moments in amplitude-modulated electric fields. Phys Rev A.

[143] Ko H, Hogan SD (2014). High-field-seeking Rydberg atoms orbiting a charged wire. Phys Rev A.

[144] Baxter C (1995). Cold Rydberg atoms as realizable analogs of Chern-Simons theory. Phys Rev Lett.

[145] Zhang JZ (1996). Angular momentum of supersymmetric cold Rydberg atoms. Phys Rev Lett.

[146] Zhang JZ (2004). Testing spatial noncommutativity via Rydberg atoms. Phys Rev Lett.

[147] Zhelyazkova V, Hogan SD (2015). Rydberg-Stark states in oscillating electric fields. Mol Phys.

[148] Reinhard A, Liebisch TC, Knuffman B, Raithel G (2007). Level shifts of rubidium Rydberg states due to binary interactions. Phys Rev A.

[149] Vogt T, Viteau M, Chotia A, Zhao J, Comparat D, Pillet P (2007). Electric-field induced dipole blockade with Rydberg atoms. Phys Rev Lett.

[150] Vliegen E (2006). Rydberg states in atom and molecule optics. PhD thesis.

[151] Seiler Ch, Hogan SD, Merkt F (2012). Dynamical processes in Rydberg-Stark deceleration and trapping of atoms and molecules. Chimia.

[152] Hogan SD (2012). Cold atoms and molecules by Zeeman deceleration and Rydberg-Stark deceleration.

